# EXcellence and PERformance in Track and Field (EXPERT)—A Mixed-Longitudinal Study on Growth, Biological Maturation, Performance, and Health in Young Athletes: Baseline Results (Part 2)

**DOI:** 10.3390/jfmk11010061

**Published:** 2026-01-30

**Authors:** Teresa Ribeiro, José Maia, Filipe Conceição, Adam Baxter-Jones, Eduardo Guimarães, Olga Vasconcelos, Cláudia Dias, Carla Santos, Ana Paulo, Pedro Aleixo, Pedro Pinto, Diogo Teixeira, Sérgio Ramos, Luís Miguel Massuça, Sara Pereira

**Affiliations:** 1 Centre of Research, Education, Innovation and Intervention in Sport (CIFI2D), Faculty of Sport, University of Porto, 4200-450 Porto, Portugal; mtsr_teresa@hotmail.com (T.R.); filipe@fade.up.pt (F.C.); eguimaraes@fade.up.pt (E.G.); olgav@fade.up.pt (O.V.); cdias@fade.up.pt (C.D.); carlass@fade.up.pt (C.S.); luis.massuca@ulusofona.pt (L.M.M.); sarasp@fade.up.pt (S.P.); 2 College of Kinesiology, University of Saskatchewan, Saskatoon, SK S7N 5B5, Canada; baxter.jones@usask.ca; 3 Center for Sport, Physical Education, Exercise and Health (CIDEFES), Universidade Lusófona, 1749-024 Lisbon, Portugal; ana.paulo@ulusofona.pt (A.P.); pedro.aleixo@ulusofona.pt (P.A.); pedro.pinto@ulusofona.pt (P.P.); p5128@ulusofona.pt (D.T.); p4886@ulusofona.pt (S.R.); 4 Portuguese Athletics Federation (FPA), 2799-538 Lisbon, Portugal; 5 Police Research Center, Higher Institute of Police Sciences and Homeland Security (IPCOL), 1349-040 Lisbon, Portugal

**Keywords:** young track and field athletes, growth and development, motor performance and psychological factors, club contexts

## Abstract

**Background**: The athletic potential of young athletes is shaped by individual and environmental factors. **Objectives**: This study examines the physical growth, body composition, biological maturation, motivation, perseverance, physical performance and contextual factors of young male and female track and field athletes. **Methods**: A total of 425 (224 girls) track and field athletes were recruited and divided into five age cohorts (10, 11, 12, 13, and 14 years respectively). Measurements were assessed across (i) individual (anthropometry, body composition, biological maturation, motivation, and perseverance), (ii) performance (motor performance), and (iii) club context domains. Data analysis used descriptive statistics for clubs’ characteristics, a two-factor ANOVA for anthropometry, body composition, biological maturation, and performance and an ANCOVA for motivation and perseverance. All analyses used STATA 18.0. **Results**: Sex-related differences were identified in physical growth, maturation, psychological, and performance variables during adolescence. Girls reached their peak height velocity (PHV) around 12 years of age, compared to 14 years in boys. At all ages (except at age 11), girls had higher body fat, and at age 12 were taller and outperformed boys in right-handgrip strength and in sprint (30 m and 40 m). From age 13 years onwards, boys became taller, with greater leg length, greater fat-free mass, and superior results (*p* < 0.05) in most performance tests. Psychologically, girls reported higher levels of interest–enjoyment, effort–importance, relatedness, and perceived choice; no sex differences were found in perseverance. The clubs involved were of small size, with developing, yet qualified, coaches, with limited support staff and infrastructure. **Conclusions**: Clear sex differences in physical growth, psychological, and performance variables emerged during adolescence, and were related in part to earlier maturation in girls. Further, there was variation in clubs’ infrastructure and staff that may potentially influence track and field athletes’ growth and development.

## 1. Introduction

This paper is the second of a two-part research series on the EXPERT study, presenting baseline results. Adolescence represents a sensitive period for athletic development [1,2,3,4,5]. To better understand how track and field athletes grow and develop, longitudinal studies are necessary to distinguish the effects of training from those of normal growth and maturation. However, most available data come from cross-sectional studies that focus exclusively on individual characteristics [6,7,8,9] and are unable to identify the independent relationships of growth, development, and environmental influence. For example, regarding anthropometry and body composition, Malina [6] examined track and field athletes of both sexes, from two age groups (11–13 and 14–15 years), and showed that boys were taller and heavier than girls from age 14 years onwards, whereas girls had higher percentage of body fat in both age groups, potentially reflecting differences in maturation between the sexes. Similarly, Yanci et al. [7] examined physical growth by age category (U14 and U16) and sex, and found that U16 track and field athletes were, on average, taller and heavier than those in the U14 group, with boys being taller and heavier than girls. In addition, Freitas et al. [8], profiling Brazilian U16 female track and field athletes, provided reference data for height, sitting height, leg length, and body mass. A similar trend in terms of sex differences was also reported by Zhao and Zhao [9] in their study, specifically focused on young throwers, aged 14–18 years.

Motor performance data from previous studies show consistent sex differences. For example, Malina’s study [6] found that boys outperformed girls in different age groups (11–13 and 14–15 years), in the handgrip strength, 20 m sprint, standing long jump, and 2 kg seated medicine ball throw tests. Similarly, Yanci et al. [7] reported that older track and field athletes (U16) ran faster in the 15 m sprint, and that boys outperformed girls in the countermovement jump. Also, Zhao and Zhao [9] identified sex differences in sprint performance (30 and 60 m) and explosive strength among young throwers aged 14–18 years, with boys typically showing better results than girls. Additionally, Freitas et al. [8] provided performance results for girls only, aged 13–15 years, in the 60 m sprint, shot put, long jump, and 800 m, with performance outcomes generally increasing with increasing age.

Although cross-sectional evidence provides valuable insight into physical growth and performance, it offers only a partial view of track and field athletic development. For a more encompassing understanding of young athletes’ developmental trajectories, it is also necessary to consider maturational, psychological and environmental factors, as they play important roles in this process [10,11,12]. Psychological traits, such as motivation and perseverance, are key elements in long-term athlete development of young track and field athletes [13,14]. For example, Barić et al. [13] evaluated the motivation of beginner track and field athletes aged 11–15 years and observed sex differences, with girls showing greater interest-enjoyment and boys reporting higher pressure-tension. Regarding grit, evidence within track and field is still limited, particularly in youth, but from the limited studies, results suggest that this trait may vary across athlete characteristics and the competitive context. Sigmundsson et al. [15] found sex differences among adolescents (13–19 years old), with boys scoring significantly higher. Similarly, Criticos et al. [16] assessed university track and field student-athletes specializing in field throwing (aged 18–25 years) and found differences between sexes, with males exhibiting higher average grit scores. Beyond sex-related patterns, grit has also been examined in athletic contexts across competitive levels and event categories; for instance, Ueno et al. [17] reported associations between grit and competitive level in Japanese athletes, including track and field disciplines. Complementary evidence from the endurance running context, which shares the long-term training demands characteristic of athletics, further supports the relevance of grit for sustained engagement and performance under high training loads [18,19]. In contrast, findings from other sports remain mixed (e.g., swimming and diving [20]; other sports [21,22,23]), highlighting the importance of strengthening sport-specific evidence in track and field whenever possible.

Environmental factors, namely, coaching characteristics and club structure, are essential for optimizing athletic development [24,25,26]. For example, it is expected that the quality of the coach–athlete relationship may enhance athletes’ psychological well-being [27], with successful track and field coaches of national-level athletes being typically older, more experienced, and themselves being former high-level athletes [25]. Additionally, clubs contribute significantly to athletes’ sustainable talent development by providing a structured, supportive, and well-equipped environment that addresses athletes’ social, physical, and psychological needs [26].

Notwithstanding the relevance of previous reports, to the best of our knowledge, no comprehensive cross-sectional or longitudinal studies have been conducted on young track and field athletes encompassing their physical growth, body composition, biological maturation, motivation, perseverance, and physical performance, together with information on their support systems, that is, coaches and clubs.

This paper presents part of the baseline results of the EXPERT mixed-longitudinal study, and its aims are (i) to describe the physical growth, body composition, biological maturation, motivation, perseverance, and motor performance of young female and male track and field athletes, as well as the characteristics of their clubs, and (ii) to analyze age- and sex-differences, including age-by-sex interactions, in physical growth, body composition, biological maturation, motivation, perseverance, and motor performance. This novel information is expected to be of interest to track and field researchers until longitudinal data on youngsters’ physical growth and development, as well as their environments, can be provided.

## 2. Materials and Methods

### 2.1. Design, Sample, and Procedures

Data presented in this paper comes from a larger research project entitled Excellence in Performance and Health: A developmental study in young track and field athletes (EXPERT). A detailed description of the EXPERT study is available elsewhere [28]. In brief, the study was conducted in two Portuguese cities, Porto and Lisbon, involving two research centers: the Centre of Research, Education, Innovation, and Intervention in Sport (CIFI2D) from the Faculty of Sport of the University of Porto (FADEUP), and the Research Center in Sports, Physical Education, and Exercise and Health (CIDEFES) from the Faculty of Physical Education and Sports of the Lusófona University. National and local sporting institutions also collaborated as stakeholders, including the Portuguese Athletics Federation, the Portuguese Association of Athletics Coaches, and the Athletics Associations of Porto and Lisbon, along with their clubs. EXPERT’s main goal was to examine the dynamic relationship between the individual characteristics of young track and field athletes and multiple environmental factors influencing their motor performance and health-related traits.

Data collection for the EXPERT study is still ongoing. The sample size was calculated a priori, and detailed information regarding this calculation is provided in Ribeiro et al. [28]. In this paper, baseline data from the individual, performance, and environmental domains are reported. A total of 425 track and field athletes (224 girls and 201 boys), aged between 10 and 14 years, were assessed at study entry. Rigorous measurement protocols and quality control procedures were implemented. Informed consent was obtained from parents or legal guardians, with approval from the Ethics Committee of the lead institution (CEFADE 38.2023).

### 2.2. Measurements

Following the EXPERT design, measurements were clustered into three study domains: individual, performance, and environmental.

#### 2.2.1. Individual Domain

In the individual domain, data related to physical growth and body composition, biological maturation, motivation, and perseverance were collected. Physical growth and body composition data were collected in accordance with the standards of the International Working Group on Kinanthropometry protocols [29]. Measurements included height, sitting height, and leg length, with the average of two closest measurements used for analysis. Body mass, body fat, and fat-free mass were measured using a portable bioimpedance scale (Tanita BC-553). All athletes were assessed while wearing light clothing, without shoes, and with metallic objects removed. The Tanita BC-553 device automatically applies the paediatric equation to estimate body fat percentage and fat-free mass. Biological maturation was estimated using the maturity offset methodology [30], which indicates the temporal distance (in years) from the occurrence of an individual’s peak height velocity (PHV), either prior to (−) or after (+).

Motivation and perseverance were assessed using two questionnaires administered to athletes aged 12 and older [31,32]. The Intrinsic Motivation Inventory [33], adapted and validated for the Portuguese population [31], measured levels of interest-enjoyment, perceived competence, effort-importance, and pressure-tension. The relatedness and perceived choice [33] were also incorporated and adapted for this study. Athletes answered 33 items using a Likert scale ranging from 1 (totally disagree) to 5 (totally agree). The Short Grit Scale (Grit-S) was used to assess perseverance and sustained interest in long-term goals, capturing dimensions of perseverance of effort (4 items) and consistency of interest (4 items) [32]. In this study, the Portuguese version [34] was used, which retained seven items (perseverance of effort: 3; consistency of interest: 4). Athletes answered on a 5-point Likert scale, ranging from 1 (strongly disagree) to 5 (strongly agree).

#### 2.2.2. Motor Performance Domain

For the motor performance domain, several tests were employed. Static strength was measured using a handgrip dynamometer (Takei Digital Grip Strength Dynamometer Model T.K.K.5401, Takei Scientific Instruments, Tokyo, Japan). The athletes performed maximal strength (kgf) for 5 s. The test result was defined as the best performance achieved in two trials for both hands [35]. Running speed was measured using a 40 m sprint assessed by a photoelectric cell system, Speed Trap II (Brower Timing Systems LLC., Draper, UT, USA). Time (s) was also registered at 5, 10, 20, 30, and 40 m splits. Athletes performed two trials each, and the best result was considered [36]. Abdominal muscle strength and endurance were assessed using the one-minute sit-up test, in which athletes performed as many repetitions as possible in a single trial [37]. Upper body explosive strength was assessed with two tests: the seated medicine ball throw [38] and the seated single-arm shot put throw [39] for both hands/sides. In both tests, the athletes were seated against a wall (zero measurement line) and threw a 3 kg medicine ball (23 cm in diameter) forwards, with the distance measured at the landing point. Each athlete performed three throws per test, and their best trial (distance in m) was recorded. Lower body explosive strength was assessed through the standing long jump (cm) [40] and three vertical jump tests (squat jump [41], countermovement jump [41], and 10/5 repeated jump test [42]), with the performance of the vertical jumps (cm) recorded using a Chronojump Boscosystem mat (Chronojump System, Barcelona, Spain). In the standing long jump, squat jump, and countermovement jump tests, the best of three repetitions was used as the test result. In the 10/5 repeated jump, only one trial was performed [43]. The RSI score—an index used to assess the neuromuscular system’s reactive and elastic capacity during jumping—was calculated as the average of the best five jumps with the best RSI. Finally, the aerobic component was assessed using the 1000 m test [44] on a 400 m track, where athletes were instructed to cover the distance as fast as possible—running or walking if needed—with results recorded in seconds (Geonaute Watch Chrono 700, Shanghai, China). This test is more specific to track and field than other commonly used tests (e.g., shuttle run) since it involves continuous linear running without changes of direction.

#### 2.2.3. Club Domain

Club information was obtained using a questionnaire completed by the club director covering four domains: (i) club characterization (number of sports, number of track and field athletes, and number of years of the club’s track and field section), (ii) human resources (number of coaches per club, coaches’ level category certification, and staff), (iii) club infrastructures (own facilities, practices always in club’s facilities, complementary equipment, and accessible by public transport), and (iv) club communication (communications manager, social media, flyers/publicity, and radio station or tv/online channel). Club directors provided responses to multiple-choice questions (single- or multiple-selection), binary questions (yes/no), and open-ended questions. This comprehensive approach gave insight into the social and organizational context influencing athletes’ well-being and training.

### 2.3. Data Quality Control

To ensure data quality, several procedures were implemented. First, the team was trained and certified by senior study members to perform all measurements. Second, a pilot study was conducted prior to the main study to ensure the quality of data collection procedures. Third, throughout data collection, a random sample of track and field athletes was re-evaluated to ensure in-field reliability. Fourth, reliability estimates were calculated, with technical error of measurement (TEM) values of 0.03 cm for height, 0.01 cm for sitting height, 0.06 cm for leg length, 0.01 kg for body mass, 0.28% for body fat, and 0.07 kg for fat-free mass. The one-way random effects ANOVA-based intraclass correlation values for physical performance tests ranged from 0.83 (sit-up test) to 0.99 (40 m sprint and standing long jump tests). Finally, the data was cleaned to correct for possible errors in data entry and putative outliers.

### 2.4. Statistical Analysis

Basic exploratory checks ensured that data quality was as advocated: systematic data entry error detection procedures, outlier identification methods, Kolmogorov–Smirnov tests for assessing normality, and descriptive statistics. For physical growth and body composition, biological maturation, and motor performance domains, a two-factor ANOVA model was used to test differences between sexes and ages, as well as age-by-sex interaction effects. Mean estimates were stratified by sex and age, and detailed contrasts of marginal linear predictions were used to better understand differences between groups. Given the large number of statistical tests performed, *p*-values were adjusted for multiple comparisons using the Bonferroni correction. For motivation and perseverance characteristics, data were analyzed only from athletes aged 12 years and older, as previously recommended [31,32]. For this, an ANCOVA was used to compare the mean scores of boys and girls, adjusted for their chronological age. The omega squared (ω^2^) was computed as a measure of effect size, because it has less bias (and bias correction provides more accurate effect sizes), it is better for generalization (compared to, for example, eta squared), and allows a more honest reporting (given the avoidance of overstated results); it was interpreted as small (≤0.059), moderate (0.060 to 0.139), or large (≥0.140) [45,46]. Finally, for the club context, results are presented using basic descriptive statistics. All analyses were conducted in STATA version 18 [47].

The broader project follows a mixed-longitudinal design and, beyond using the triple logistic model to describe physical growth [48], also uses the triple logistic model to describe physical growth [48]. A hierarchical linear growth model is used [49,50] to mirror the data structure: repeated observations (level I) nested within subjects (level II), which are nested within clubs (level III). Furthermore, as advocated [51,52], data will be aligned by age at peak height velocity to identify sensitive periods for motor performance.

## 3. Results

### 3.1. Anthropometry, Body Composition, and Biological Maturation

Descriptive statistics for young track and field athletes by age and sex are presented in Table 1. A visual representation of the two-factor ANOVA results for anthropometry and body composition is provided in Supplementary File S1. A significant main effect of age on all anthropometric and body composition variables was observed (except for body fat), with progressive increases in height (F = 109.46; *p* < 0.001; ω^2^ = 0.51), sitting height (F = 40.05; *p* < 0.001; ω^2^ = 0.27), leg length (F = 58.45; *p* < 0.001; ω^2^ = 0.36), and body mass (F = 49.81; *p* < 0.001; ω^2^ = 0.32). Fat-free mass also increased significantly with age (F = 80.16; *p* < 0.001; ω^2^ = 0.43), while body fat mass showed no differences across age groups (*p* > 0.05). Sex differences were observed in sitting height (F = 6.83; *p* = 0.009; ω^2^ = 0.01), leg length (F = 16.36; *p* < 0.001; ω^2^ = 0.04), body fat (F = 116.72; *p* < 0.001; ω^2^ = 0.22), and fat-free mass (F = 27.78; *p* < 0.001; ω^2^ = 0.06). Significant age-by-sex interactions were found for height (F = 7.88; *p* < 0.001; ω^2^ = 0.06), leg length (F = 5.95; *p* < 0.001; ω^2^ = 0.05), body fat (F = 10.20; *p* < 0.001; ω^2^ = 0.08), and fat-free mass (F = 7.43; *p* < 0.001; ω^2^ = 0.06). Girls were taller than boys by 12 years (+4.19 cm), but boys surpassed girls at 13 (+4.85 cm) and 14 years (+6.11 cm). Similarly, boys had greater leg length than girls at ages 13 (+4.81 cm) and 14 (+5.22 cm). Girls had higher body fat mass than boys (except at age 11), with the most pronounced differences at ages 13 (+12.00%) and 14 years (+9.18%). In contrast, boys showed significantly greater fat-free mass than girls, at ages 13 and 14 (+5.57 kg and +6.96 kg, respectively).

Finally, maturity offset differed significantly across age groups (F = 277.02; *p* < 0.001; ω^2^ = 0.72), and between sexes (F = 624.09; *p* < 0.001; ω^2^ = 0.60), with girls being more advanced in biological maturation at all ages than boys. No age-by-sex interaction was found for maturity offset (*p* > 0.05).

The magnitude of the effect size indicated that age exerted a large influence on most anthropometric and body composition variables (0.27–0.51), whereas sex and age-by-sex interaction effects were generally small to large (0.01–0.22 and 0.05–0.08, respectively). Despite statistical significance, interaction patterns accounted for a limited proportion of variance compared to the main effect of age.

Overall, height, sitting height, leg length, and body mass increased with age in both boys and girls. At the age of 12, girls were slightly taller than boys, reflecting their earlier biological maturation. However, from age 13 onward, boys began to surpass girls in height, showing larger gains in fat-free mass that coincided with their growth spurt. These results underscore the importance of considering both chronological age and biological maturation when examining sex differences in body size and body composition in young athletes. Earlier maturation in girls at age 12 accounts for their temporary height advantage, whereas the subsequent growth spurt in boys, from age 13, aligns with their greater gains in height, leg length, and fat-free mass. These findings underscore the importance of biological maturation in understanding sex-specific developmental trajectories in the motor performance and competition of youth track and field athletes.

### 3.2. Motivation and Perseverance

Table 2 presents the results on motivation and perseverance. The questionnaires assessing motivation and perseverance were administered to athletes aged 12 years and older. This age threshold was defined because younger athletes showed difficulties in comprehension and independent response, and the instruments used (e.g., IMIp) were developed for adolescents and adults. For this reason, the initial sample of 425 athletes was reduced to 128. Within the IMIp, the ANCOVA findings showed that compared to boys, girls reported higher levels of interest-enjoyment (F = 4.59; *p* < 0.034; ω^2^ = 0.03), effort-importance (F = 4.51; *p* < 0.036; ω^2^ = 0.03), relatedness (F = 4.13; *p* < 0.044; ω^2^ = 0.02), and perceived choice (F = 10.20; *p* < 0.002; ω^2^ = 0.07). No significant sex differences were found for perceived competence and pressure-tension (*p* > 0.05). Regarding GRIT-S, no statistically significant differences were observed in both consistency of interest and perseverance of effort items (*p* > 0.05).

Although several sex differences in IMIp dimensions reached statistical significance, the associated effect sizes were small to moderate (0.02–0.07), indicating that these differences, while consistent, may not be practically relevant given the multifactorial nature of growth, maturation, and motivation in youth athletes. The corresponding effect sizes suggest that sex explained a small proportion of the variance in most motivational dimensions, with a moderate effect observed for perceived choice, indicating a more meaningful sex-related difference for this psychological construct.

No significant effects of age were observed in IMIp and GRIT-S (*p* > 0.05).

### 3.3. Motor Performance

Descriptive statistics for young track and field athletes by age and sex are shown in Table 3. A visual representation of the two-factor ANOVA results for motor performance tests is in Supplementary File S2.

Athletes showed significant age-related improvements across all motor performance variables. Large age effects were observed in strength-related measures, including right-hand handgrip (F = 63.03, *p* < 0.001, ω^2^ = 0.38), left-hand handgrip (F = 55.16, *p* < 0.001, ω^2^ = 0.35), and the 3 kg seated medicine ball throw (F = 77.96, *p* < 0.001, ω^2^ = 0.43), including right-arm (F = 73.31, *p* < 0.001, ω^2^ = 0.41) and left-arm throws (F = 63.68, *p* < 0.001, ω^2^ = 0.38). Moderate to large age effects were also observed for sprint performance over 5 m (F = 17.32, *p* < 0.001, ω^2^ = 0.14), 10 m (F = 27.46, *p* < 0.001, ω^2^ = 0.20), 20 m (F = 38.00, *p* < 0.001, ω^2^ = 0.26), 30 m (F = 45.27, *p* < 0.001, ω^2^ = 0.30), and 40 m (F = 49.22, *p* < 0.001, ω^2^ = 0.33). For jumping performance, moderate to large age effects were found in the standing long jump (F = 29.42, *p* < 0.001, ω^2^ = 0.22), squat jump (F = 12.07, *p* < 0.001, ω^2^ = 0.12), and countermovement jump (F = 12.48, *p* < 0.001, ω^2^ = 0.12). Smaller age effects were observed for sit-ups (F = 9.62, *p* < 0.001, ω^2^ = 0.08), RSI (F = 2.97, *p* = 0.020, ω^2^ = 0.02), and the 1000 m run (F = 6.09, *p* < 0.001, ω^2^ = 0.06).

Sex differences were found in 5 m sprint (F = 7.16, *p* = 0.008, ω^2^ = 0.01), sit-ups (F = 13.00, *p* < 0.001, ω^2^ = 0.03), 3 kg seated medicine ball throw (F = 18.50, *p* < 0.001, ω^2^ = 0.04), 3 kg seated medicine ball throw from the right arm (F = 24.33, *p* < 0.001, ω^2^ = 0.05), 3 kg seated medicine ball throw from the left arm (F = 15.23, *p* < 0.001, ω^2^ = 0.03), standing long jump (F = 9.95, *p* = 0.002, ω^2^ = 0.02), squat jump (F = 4.93, *p* = 0.027, ω^2^ = 0.01), countermovement jump (F = 4.50, *p* = 0.035, ω^2^ = 0.01), and 1000 m run (F = 16.13, *p* < 0.001, ω^2^ = 0.05). All sex-related effects were small in magnitude.

A significant interaction effect age-by-sex was observed for handgrip right (F = 6.91, *p* < 0.001, ω^2^ = 0.06), handgrip left (F = 7.10, *p* < 0.001, ω^2^ = 0.06), 10 m sprint (F = 3.30, *p* = 0.011, ω^2^ = 0.02), 20 m sprint (F = 4.70, *p* = 0.001, ω^2^ = 0.03), 30 m sprint (F = 7.27, *p* < 0.001, ω^2^ = 0.06), 40 m sprint (F = 5.56, *p* < 0.001, ω^2^ = 0.04), 3 kg seated medicine ball throw (F = 6.97, *p* < 0.001, ω^2^ = 0.05), 3 kg seated medicine ball right arm throw (F = 3.06, *p* = 0.017, ω^2^ = 0.02), 3 kg seated medicine ball left arm throw (F = 3.17, *p* = 0.014, ω^2^ = 0.02), standing long jump (F = 5.00, *p* < 0.001, ω^2^ = 0.04), squat jump (F = 5.09, *p* < 0.001, ω^2^ = 0.05), countermovement jump (F = 3.83, *p* = 0.005, ω^2^ = 0.03), and 1000 m run (F = 3.19, *p* = 0.014, ω^2^ = 0.03). These interaction effects were small to moderate in magnitude. At age 12, girls outperformed boys in handgrip right (+2.81 kgf), but by age 14, boys surpassed girls in both right- and left-handgrip (+5.75 kgf and +6.45 kgf, respectively). Girls at age 12 are faster than boys over 30 m and 40 m sprint (−0.22 s and −0.27 s, respectively). However, at age 13, boys are faster than girls over 10 m (−0.07 s), and at age 14, boys are faster than girls at 20 m, 30 m, and 40 m (−0.21 s, −0.34 s, and −0.36 s, respectively). Boys aged 13 and 14 threw farther than girls in the 3 kg seated medicine ball throw (+0.38 m and +0.61 m, respectively): right arm (+0.31 m and +0.50 m, respectively) and left arm (+0.27 m and +0.40 m, respectively). In the standing long jump, boys aged 13 and 14 outperform girls (+19.5 cm and +16.5 cm, respectively). In vertical jumps, at ages 13 and 14, boys jump higher than girls in the squat jump (+3.69 cm and +4.98 cm, respectively), but in the countermovement jump, boys outperformed girls only at age 13 (+3.66 cm). Finally, at 1000 m, boys complete the distance faster than girls at ages 13 and 14 (−47.71 s and −43.90 s, respectively).

In summary, the results indicate that strength-related measures exhibited the largest age effects and the most consistent age-by-sex interactions, reflecting pronounced developmental gains across adolescence. Speed and jump performance showed moderate to large age effects, with sex-related differences becoming more evident at older ages. In contrast, endurance performance demonstrated smaller age effects, although boys consistently outperformed girls from early adolescence onward.

### 3.4. Club Information

The characteristics of clubs are shown in Table 4. There was considerable variation in sports modalities across clubs. For track and field, the median number of athletes per club was 96 (IQR = 72.5). The median number of years since the sport was established at the club was 29 years. Each club employed approximately six coaches, with most still attending the Level I certification course. Very few clubs reported having a physician (8.33%) or psychologist (8.33%). Half of the clubs employed a physiotherapist (50.00%), and fewer had a massage therapist (41.67%) and even fewer a nutritionist (16.67%).

Regarding infrastructure, 41.67% of the clubs owned the facilities, and 66.67% had a gym; however, fewer reported having a warm-up area (29.17%) or a clubhouse/video room (37.50%). Public transport access was available for 62.50% of clubs. All clubs used social media (100.00%), while fewer relied also on flyers (66.67%) or radio/online channels (16.67%).

These infrastructural limitations may influence training quality and athlete development, particularly in youth pathways, while also constraining clubs’ capacity for growth and sustainability.

## 4. Discussion

This study reports baseline results from the ongoing EXPERT mixed-longitudinal study of young track and field athletes (10–14 years), specifically focusing on anthropometry, body composition, biological maturation, motivation, perseverance, and motor performance, as well as their club environments at baseline. Beyond providing descriptive baseline data, this study also examines how age, sex, and their interaction influence physical, psychological, and performance characteristics.

### 4.1. Anthropometry, Body Composition, and Biological Maturation

The results showed clear age-related increases in anthropometric measures, including height, sitting height, leg length, and body mass, for both sexes, consistent with expected patterns of physical growth and biological maturation during childhood and adolescence [53]. Girls were taller at age 12, but boys surpassed them from age 13 onwards, with this difference becoming more pronounced by age 14 [6,9]. Sitting height increased with age, but with small sex differences and no age-by sex interaction effects, in line with earlier findings [54]. In contrast, boys exhibited greater leg length at ages 13–14, highlighting the contribution of lower-limb growth to the male adolescent growth spurt [55,56,57]. These findings align with established growth trajectories, in which peak height velocity occurs earlier in girls but is more prolonged and pronounced in boys during mid-adolescence [53,58]. Body mass increased with age, without significant sex differences, following the expected age-related changes in young athletes [54]. However, some studies report higher body mass in boys around age 14 [6,7,9]. These discrepancies may be explained by differences in sample characteristics and variation in maturity status. No age-related differences were observed in body fat percentage, but were consistently higher in girls, particularly at 13–14 years, in line with previous findings [6]. In contrast, fat-free mass increased with age, especially in boys, reflecting sex-specific adaptations during adolescence [53,59,60]. This pattern reflects, among other factors, the effects of estrogen on female subcutaneous fat deposition and the anabolic effects of insulin-like growth factor-1 (IGF-1), growth hormone, and testosterone during the male growth spurt [53]. The significant age-by-sex interaction indicates that sex differences in body composition become more pronounced during mid-adolescence.

Lastly, maturity status (maturity offset from PHV) revealed that girls were biologically more advanced than boys across all age groups, with no significant age-by-sex interaction, which explains their early physical growth advantage and higher relative body fat during early adolescence, before being surpassed by boys from 13 years onwards. Overall, these findings highlight the importance of considering chronological age, sex, and maturity status when interpreting anthropometric and body composition data in adolescents.

### 4.2. Motivation and Perseverance

Regarding motivation, girls reported higher levels of interest-enjoyment, effort-importance, relatedness, and perceived choice in track and field practice. Conversely, no statistically significant sex differences were found in the subscales of perceived competence and pressure-tension. These findings are in partial agreement with previous reports. On the one hand, they corroborate evidence that girls tend to be more motivated in contexts that emphasise collaboration and social relationships, nurturing the development of competence and relatedness [61,62]. Barić et al. [13] also identified higher levels of interest-enjoyment among girls engaged in beginner track and field, whereas boys reported greater pressure-tension. On the other hand, the results diverge from studies that reported higher scores among boys in perceived competence, effort-importance, and interest-enjoyment compared with girls, the difference being that these studies were conducted in general population samples within the context of physical education classes [63]. Such discrepancies may reflect the distinct ways in which boys and girls achieve their basic psychological needs, as proposed by the Self-Determination Theory [64], as well as the influence of contextual and cultural factors specific to each learning or training environment. Moreover, in the present study, no significant differences were observed between sexes in perceived competence and pressure-tension, both of which showed relatively low values, suggesting that these dimensions may be more influenced by the training context than by biological sex itself. Since pressure-tension is recognized as a negative aspect of motivation [31], the similar scores observed in boys and girls may indicate that the sporting environment provided a balanced level of challenge and emotional support for both sexes. In summary, these results support the view that sex-related motivational differences are not absolute and are largely dependent on the motivational climate and contextual factors of each environment. [65]. Therefore, creating an environment that supports the satisfaction of athletes’ basic psychological needs appears fundamental to promoting intrinsic motivation and, consequently, enhancing sports performance [66,67].

With regards to perseverance (grit), the results showed no significant differences between boys and girls, supporting findings from studies involving fencers [21] and athletes from various other sports [22,23]. Although Cain et al. [20] reported sex differences in swimmers and divers, with higher scores among females, these findings appear context-specific and cannot be generalized. Furthermore, higher levels of grit among competitive athletes than among recreational or non-athletes [22,68] reinforce the notion that perseverance, rather than innate talent, is a critical factor distinguishing more successful young athletes [12,69,70].

### 4.3. Motor Performance

The results showed significant age-related improvements across all motor performance tests, reflecting the expected performance patterns in children and adolescent athletes [53]. Systematic increases with age and maturation were observed in handgrip strength (both hands), sprint performance (5–40 m), sit-ups, medicine ball throws (both arms and each arm, right and left), horizontal and vertical jumps, and 1000 m run. Moreover, sex-related differences were identified in almost all motor components. These results reflect the typical divergence between the sexes following the onset of puberty, when boys experience prolonged linear growth, increased muscle mass, and, consequently, greater muscular strength than girls. In contrast, girls reach peak height velocity earlier and accumulate a higher proportion of subcutaneous fat [53]. More importantly, the significant age-by-sex interaction for handgrip strength, sprints, medicine ball throws, jumps, and the 1000 m run demonstrates that differences between boys and girls become increasingly pronounced during mid-adolescence. For example, in handgrip strength, 12-year-old girls showed higher values in the right hand, whereas 14-year-old boys outperformed girls in both hands. This finding aligns with Malina et al. [6], who reported a male advantage from age 12, whereas Lesinski et al. [54] also identified age- and sex-related differences at a later stage, possibly reflecting methodological variations in testing protocols. In sprints, results showed that 12-year-old girls outperformed boys over 30 m and 40 m, whereas by ages 13, boys began to surpass girls over 10 m and at ages 14 over 30 m and 40 m, reflecting the impact of physical growth, hormonal changes, and increased muscle mass. These findings are partially supported by previous studies, where Yanci et al. [7] found no sex differences in shorter distances (5 m and 15 m), whereas Malina et al. [6] and Zhao and Zhao [9] reported a male advantage at 20 m and 30 m, respectively, from early adolescence. For upper-limb explosive strength, assessed by the medicine ball throw (both arms, right arm, and left arm), boys aged 13–14 threw farther than girls, confirming the influence of muscle mass and explosive strength on performance. These findings are partially supported by Malina et al. [6], who also reported a male advantage in the seated medicine ball throw, although at an earlier stage (ages 11–15). Such discrepancies may be attributable to methodological differences, particularly the use of a 2 kg ball in their protocol, compared with the 3 kg ball used in the presented study. In the jump tests, boys outperformed girls at ages 13 and 14 (except for the countermovement jump, in which they only surpassed at age 13). These trends are consistent with those reported by Malina et al. [6] and Zhao and Zhao [9], who found that boys outperform girls during adolescence. However, Yanci et al. [7] reported no differences in U14 and U16 track and field athletes. Temfemo et al. [71] found significant sex differences in squat jump already at age 12, in contrast with this study, possibly due to the inclusion of non-athlete participants. Lesinski et al. [54] also reported sex- and age-related differences in countermovement jump, and Yanci et al. [7] found sex differences favouring boys. In the RSI test, only age-related improvements were observed, contrasting with the findings of Lehnert et al. [72], who reported sex differences (but only in the U16 category). In aerobic performance, few studies have examined age- and sex-related differences in the 1000 m run among young track and field athletes. Chatzakis et al. [44] showed that performance is influenced by a combination of aerobic (VO_2max_ and vVO_2max_) and anaerobic (CMJ) factors, as well as anthropometric factors (body mass and body fat percentage). Moderate negative correlations between CMJ and 1000 m run time indicate that greater lower-limb explosive strength is associated with faster performance, despite the predominantly aerobic nature of the event. Boys exhibited higher CMJ values than girls at ages 13–14, partially explaining their faster 1000 m run times. Clear sex differences in body composition were observed, with girls showing higher body fat and boys greater fat-free mass at these ages. These findings align with those of Chatzakis et al. [44], showing that higher body mass and body fat percentage are negatively associated with 1000 m running times. Together, the results suggest that sex differences in adolescent 1000 m performance are largely driven by maturational changes in body composition and strength rather than chronological age alone.

These findings are consistent with growth patterns described in the literature [53]. During the pubertal growth spurt, boys experience greater increases in lean mass and muscle strength under the influence of insulin-like growth factor 1 (IGF-1), growth hormone, and testosterone, whereas girls show higher fat deposition induced by estrogen [73,74]. Since physical performance is related to muscle strength [75,76], and strength is associated with the size and body mass of athletes as well as their body composition [77,78], taller and heavier athletes with greater lean mass tend to achieve better performance. In summary, the performance advantage observed in boys in muscular strength, sprinting, jumping, and endurance running from the age of 13 reflects not only hormonal influences but also their biological maturation status and physical growth changes, as previously described.

From a developmental perspective, the magnitude of the age effects suggests that not all motor performance tests are equally linked to physical growth and biological maturation. Variables closely related to maximal and explosive strength (handgrip strength, medicine ball throws, and horizontal jumping) as well as linear speed (sprint performance over 20 to 40 m) showed moderate to large effect sizes, indicating high sensitivity to age-related biological changes. In contrast, capacities associated with neuromuscular reactivity and endurance, including the reactive strength index (RSI), sit-ups, and the 1000 m run, showed smaller effect sizes, suggesting greater consistency across age groups. These findings highlight a differentiated pattern of motor performance development, in which strength- and power-related tests are more strongly influenced by physical growth and biological maturation than reactive or endurance-related tests.

### 4.4. Club Information

The results indicate that the track and field clubs participating in this study are relatively small organizations, with a median of 96 athletes per club, and typically offer only one sport. Human resources are limited: clubs employ an average of six coaches, most of whom are attending or have completed Level I or II certification, but multidisciplinary support staff such as physicians (8.33%), psychologists (8.33%), nutritionists (16.67%), or physiotherapists (50.00%) are scarce. Infrastructure is also constrained; although most clubs have access to a gym (66.67%), only a minority possess specialized spaces such as warm-up areas (29.17%), clubhouses (37.50%), or medical/physiotherapy offices (54.17%). Clubs maintain an active presence on social media, but alternative communication channels (flyers, radio, online platforms) are infrequently used, suggesting opportunities for improvement.

In summary, the results suggest that the track and field clubs participating in this study are relatively small and multifunctional organizations, with developing and qualified coaches but limited support staff and infrastructure compared to those in more popular sports. These findings reinforce the importance of private/public policies and strategies aimed at the professionalization of clubs, including the recruitment of multidisciplinary teams, improvements to facilities, and the expansion of communication and promotion channels. Investment in these dimensions may not only improve the quality of support offered to athletes but also strengthen the sustainability and attractiveness of sports organizations.

### 4.5. Limitations

Notwithstanding the importance of this study has limitations that must be acknowledged: (i) The track and field athletes assessed were between 10 and 14 years old, which only captures a specific, but very important stage of their development; (ii) although the importance of longitudinal data was mentioned, only baseline data was provided, i.e., cross-sectional that may still be of interest to researchers in the field; (iii) the analyses adjusted only for age and sex, and did not consider potential confounding variables such as training volume, sport specialization, and training age; (iv) given that the psychological variables were only answered by 128 athletes aged 12+, for reasons linked to comprehension and interpretability of all questions, this may limit, therefore, the representativeness and generalizability for younger age groups; (v) the structure and content of the questionnaire employed to assess club characteristics was exploratory in nature as well as study-specific, potentially limiting the generalizability and comparability of these findings; (vi) in addition, although data were collected from most youth training track and field clubs in the two major Portuguese cities, the reality of these clubs may not fully reflect the conditions of clubs in other cities, particularly those with fewer resources.

Irrespective of these limitations, this study has several strong points: (i) fills a gap in the literature by adopting an bioecological approach that includes both individual and environmental factors; (ii) provides fresh data on young track and field athletes; (iii) uses validated and standardized protocols, ensuring the reliability of the data covering a wide range of variables; and (iv) helps identify sensitive ages at which differences in growth, motivation, perseverance, and performance emerge between track and field boys and girls.

## 5. Conclusions

This study presents an overview of baseline characteristics of physical growth, motivation, perseverance, and motor performance in young track and field athletes, as well as a description of the clubs’ characteristics. Boys and girls exhibited different growth patterns, with girls reaching their peak height earlier and having higher fat levels, while boys grew taller and gained more lean mass during mid-adolescence. Motivation was generally higher in girls for interest-enjoyment, effort-importance, relatedness, and perceived choice, while grit was similar between sexes. Motor performance improved with age, with boys outperforming girls in strength, sprints, jumps, and running endurance from around 13 years, reflecting differences in physical growth and maturation. Regarding organizational context, the clubs have experienced coaches but limited facilities and support staff, suggesting potential constraints in available resources and professional structures. In this context, and considering the financial constraints commonly faced by sports organizations, one possible approach to addressing these challenges could be the development of partnerships with universities, volunteer programs, municipal centers, and similar institutions.

In summary, this study provides practical insights for coaches and clubs, as well as preliminary evidence that may help inform broader sports policies aimed at fostering healthy development and sustaining youth participation in track and field. Further research is needed to track athletes over time, using longitudinal data to examine their developmental pathways and to understand how performance is influenced by health status and environmental factors. This will be the next goal when the EXPERT data collection is finished.

## Figures and Tables

**Table 1:** Descriptive statistics [expected means (EMs) and standard errors (SEs)] from two-way ANOVA for athletes’ anthropometry, body composition, and biological maturation data, by cohort and sex.

Individual Domain		10 Years (C1)	11 Years (C2)	12 Years (C3)	13 Years (C4)	14 Years (C5)
		n = 59 (♀ = 22; ♂ = 37)	n = 104 (♀ = 54; ♂ = 50)	n = 99 (♀ = 56; ♂ = 43)	n = 110 (♀ = 65; ♂ = 45)	n = 53 (♀ = 27; ♂ = 26)
		EM ± SE	EM ± SE	EM ± SE	EM ± SE	EM ± SE
*Anthropometry and body composition*						
Height (cm)	Girls	141.92 ± 1.49	147.09 ± 0.95	154.33 ± 0.96	157.60 ± 0.87	159.12 ± 1.35
	Boys	141.10 ± 1.15	145.83 ± 0.99	150.14 ± 1.07	162.45 ± 1.04	165.23 ± 1.37
Sitting height (cm)	Girls	77.38 ± 1.07	78.49 ± 0.68	80.22 ± 0.67	83.02 ± 0.62	85.08 ± 0.96
	Boys	74.50 ± 0.82	75.73 ± 0.72	79.15 ± 0.77	83.06 ± 0.74	84.95 ± 1.04
Leg length (cm)	Girls	64.53 ± 1.16	68.60 ± 0.74	73.16 ± 0.73	74.58 ± 0.67	74.04 ± 1.04
	Boys	66.60 ± 0.89	69.58 ± 0.77	71.45 ± 0.84	79.39 ± 0.81	79.26 ± 1.09
Body mass (kg)	Girls	33.70 ± 1.84	39.01 ± 1.12	43.41 ± 1.10	49.22 ± 1.02	49.45 ± 1.61
	Boys	34.44 ± 1.37	38.03 ± 1.18	40.66 ± 1.26	48.49 ± 1.23	53.22 ± 1.61
Body fat (%)	Girls	22.21 ± 1.26	21.23 ± 0.80	21.58 ± 0.79	24.62 ± 0.73	24.93 ± 1.13
	Boys	17.74 ± 0.97	18.43 ± 0.84	17.22 ± 0.90	12.62 ± 0.88	15.75 ± 1.16
Fat-free mass (kg)	Girls	27.36 ± 1.11	30.37 ± 0.71	33.67 ± 0.70	36.61 ± 0.65	37.53 ± 1.00
	Boys	28.59 ± 0.86	31.05 ± 0.74	33.39 ± 0.79	42.18 ± 0.78	44.49 ± 1.02
*Biological maturation*						
Maturity offset (years)	Girls	−1.48 ± 0.13	−0.74 ± 0.08	0.09 ± 0.08	0.93 ± 0.08	1.53 ± 0.12
	Boys	−3.02 ± 0.10	−2.37 ± 0.09	−1.67 ± 0.09	−0.69 ± 0.09	0.22 ± 0.12

C1 = cohort 1; C2 = cohort 2; C3 = cohort 3; C4 = cohort 4; C5 = cohort 5.

**Table 2:** Expected means (EMs) and standard errors (SEs) from ANCOVA for motivation and perseverance of athletes aged 12 to 14, by sex.

Individual Domain		
*Motivation*		n = 128 (♀ = 70; ♂ = 58)
		EM ± SE
Interest-enjoyment	Girls	4.62 ± 0.06
	Boys	4.42 ± 0.07
Perceived competence	Girls	3.91 ± 0.08
	Boys	3.98 ± 0.08
Effort-importance	Girls	4.57 ± 0.06
	Boys	4.38 ± 0.07
Pressure-tension	Girls	3.79 ± 0.11
	Boys	3.84 ± 0.12
Relatedness	Girls	4.34 ± 0.07
	Boys	4.13 ± 0.08
Perceived choice	Girls	4.36 ± 0.07
	Boys	4.03 ± 0.08
*Perseverance*		n = 126 (♀ = 68; ♂ = 58)
		EM ± SE
Consistency of interest	Girls	3.59 ± 0.12
	Boys	3.53 ± 0.12
Perseverance of effort	Girls	4.09 ± 0.08
	Boys	4.13 ± 0.08

**Table 3:** Descriptive statistics [expected means (EMs) and standard errors (SEs)] from two-way ANOVA for athletes’ motor performance by cohort and sex.

Motor Performance Domain		10 Years (C1)	11 Years (C2)	12 Years (C3)	13 Years (C4)	14 Years (C5)
		n = 59 (♀ = 22; ♂ = 37)	n = 104 (♀ = 54; ♂ = 50)	n = 99 (♀ = 56; ♂ = 43)	n = 110 (♀ = 65; ♂ = 45)	n = 53 (♀ = 27; ♂ = 26)
		EM ± SE	EM ± SE	EM ± SE	EM ± SE	EM ± SE
Handgrip right hand strength (kgf)	Girls	16.41 ± 1.09	16.35 ± 0.72	21.25 ± 0.65	24.09 ± 0.60	23.78 ± 0.93
	Boys	15.77 ± 0.81	17.24 ± 0.70	18.45 ± 0.74	24.92 ± 0.74	29.52 ± 0.95
Handgrip left hand strength (kgf)	Girls	15.87 ± 1.06	16.15 ± 0.68	19.66 ± 0.63	22.68 ± 0.59	22.25 ± 0.91
	Boys	14.51 ± 0.79	16.54 ± 0.68	17.91 ± 0.72	22.91 ± 0.74	28.70 ± 0.93
5 m sprint (s)	Girls	1.28 ± 0.02	1.28 ± 0.01	1.23 ± 0.01	1.23 ± 0.01	1.19 ± 0.02
	Boys	1.27 ± 0.02	1.29 ± 0.02	1.21 ± 0.02	1.17 ± 0.02	1.12 ± 0.02
10 m sprint (s)	Girls	2.21 ± 0.03	2.20 ± 0.02	2.09 ± 0.02	2.10 ± 0.02	2.05 ± 0.03
	Boys	2.20 ± 0.02	2.19 ± 0.02	2.14 ± 0.02	2.03 ± 0.02	1.96 ± 0.03
20 m sprint (s)	Girls	3.90 ± 0.05	3.82 ± 0.03	3.60 ± 0.03	3.61 ± 0.03	3.57 ± 0.05
	Boys	3.85 ± 0.04	3.84 ± 0.03	3.71 ± 0.04	3.50 ± 0.04	3.36 ± 0.05
30 m sprint (s)	Girls	5.55 ± 0.07	5.38 ± 0.05	5.08 ± 0.04	5.06 ± 0.04	5.02 ± 0.06
	Boys	5.47 ± 0.05	5.39 ± 0.05	5.30 ± 0.05	4.92 ± 0.05	4.67 ± 0.07
40 m sprint (s)	Girls	7.29 ± 0.10	6.95 ± 0.06	6.58 ± 0.06	6.54 ± 0.05	6.41 ± 0.09
	Boys	7.14 ± 0.07	7.00 ± 0.07	6.85 ± 0.07	6.36 ± 0.07	6.05 ± 0.09
Sit-ups (repetitions)	Girls	24.55 ± 1.46	27.15 ± 0.95	30.16 ± 0.92	30.84 ± 0.85	28.40 ± 1.37
	Boys	29.62 ± 1.12	28.21 ± 0.99	31.00 ± 1.04	34.51 ± 1.02	30.60 ± 1.37
3 kg seated medicine ball throw (m)	Girls	2.17 ± 0.10	2.34 ± 0.06	2.67 ± 0.06	2.91 ± 0.06	3.00 ± 0.09
	Boys	2.21 ± 0.07	2.34 ± 0.07	2.63 ± 0.07	3.29 ± 0.07	3.62 ± 0.09
3 kg seated medicine ball throw right arm (m)	Girls	1.87 ± 0.10	2.12 ± 0.06	2.36 ± 0.06	2.68 ± 0.05	2.81 ± 0.08
	Boys	2.05 ± 0.07	2.19 ± 0.06	2.43 ± 0.07	2.99 ± 0.07	3.31 ± 0.09
3 kg seated medicine ball throw left arm (m)	Girls	1.88 ± 0.08	2.05 ± 0.05	2.20 ± 0.05	2.50 ± 0.05	2.58 ± 0.08
	Boys	1.91 ± 0.07	2.06 ± 0.06	2.29 ± 0.06	2.77 ± 0.06	2.98 ± 0.08
Standing long jump (cm)	Girls	143.27 ± 4.86	154.31 ± 3.19	166.76 ± 3.08	168.58 ± 2.83	172.00 ± 4.39
	Boys	150.68 ± 3.75	153.32 ± 3.33	161.70 ± 3.48	188.11 ± 3.40	188.48 ± 4.56
Squat jump (cm)	Girls	21.04 ± 1.23	21.48 ± 0.77	23.45 ± 0.72	24.02 ± 0.70	22.85 ± 1.31
	Boys	21.00 ± 0.91	21.85 ± 0.81	21.19 ± 0.86	27.71 ± 0.81	27.84 ± 1.23
Countermovement jump (cm)	Girls	21.39 ± 1.28	22.63 ± 0.81	24.29 ± 0.76	25.14 ± 0.73	23.54 ± 1.42
	Boys	21.19 ± 0.95	22.55 ± 0.86	22.87 ± 0.90	28.80 ± 0.84	28.36 ± 1.28
RSI (score)	Girls	0.89 ± 0.09	0.86 ± 0.05	0.95 ± 0.05	1.03 ± 0.05	0.96 ± 0.09
	Boys	0.79 ± 0.06	0.90 ± 0.05	0.89 ± 0.06	1.01 ± 0.06	1.03 ± 0.09
1000 m run (s)	Girls	318.19 ± 13.02	285.91 ± 7.94	277.27 ± 7.85	285.63 ± 7.94	278.35 ± 11.65
	Boys	289.58 ± 10.21	279.03 ± 8.93	279.80 ± 8.80	237.92 ± 8.45	234.45 ± 11.65

RSI = reactive strength index; C1 = cohort 1; C2 = cohort 2; C3 = cohort 3; C4 = cohort 4; C5 = cohort 5.

**Table 4:** Descriptive statistics for clubs’ data.

Clubs (n = 24)	Median (IQR)	Mean ± SD	Min–Max	n (%)
*Club characteristics*				
Number of sports within the club	1 (2.50)		1–35	
Number of track and field athletes	96 (72.50)		22–525	
Number of years of the club’s track and field section	29 (31.50)		9–118	
*Human resources*				
Number of coaches per club		5.79 ± 7.28	1–38	
Coaches’ level category certification				
Number of coaches with level I		1.71 ± 0.92	1–4	
Number of coaches with level II		2.08 ± 1.26	1–4	
Number of coaches with level III		1.11 ± 0.33	1–2	
Number of coaches attending level I		2.75 ± 1.50	2–5	
Staff				
Physician (Yes/No)				2 (8.33)/22 (91.67)
Physiotherapist (Yes/No)				12 (50.00)/12 (50.00)
Massage therapist (Yes/No)				10 (41.67)/14 (58.33)
Psychologist (Yes/No)				2 (8.33)/22 (91.67)
Nutritionist (Yes/No)				4 (16.67)/20 (83.33)
*Club infrastructure*				
Own facilities (Yes/No)				10 (41.67)/14 (58.33)
Practices always in club’ facilities (Yes/No)				0 (0.00)/24 (100.00)
Complementary equipment				
Simplified track (Yes/No)				2 (8.33)/22 (91.67)
Gym (Yes/No)				16 (66.67)/8 (33.33)
Warm-up area (Yes/No)				7 (29.17)/17 (70.83)
Medical/Physiotherapy office (Yes/No)				13 (54.17)/11 (45.83)
Clubhouse/Video room (Yes/No)				9 (37.50)/15 (62.50)
Accessible by public transport (Yes/No)				15 (62.50)/9 (37.50)
*Club communication*				
Communications manager (Yes/No)				11 (45.83)/13 (54.17)
Social media (Yes/No)				24 (100.00)/0 (0.00)
Flyers/Publicity (Yes/No)				16 (66.67)/8 (33.33)
Radio station or TV/Online channel (Yes/No)				4 (16.67)/20 (83.33)

IQR = interquartile range.

## Data Availability

The data that support the findings of this study are available from the corresponding author upon reasonable re-quest, in compliance with policies regarding data sharing at the Faculty of Sports, University of Porto, Porto, Portugal.

## Supplementary Materials

The following supporting information can be downloaded at: https://www.mdpi.com/article/10.3390/jfmk11010061/s1, Figure S1: Graphical representation of mean height values for boys and girls by age; Figure S2: Graphical representation of mean sitting height values for boys and girls by age; Figure S3: Graphical representation of mean leg length values for boys and girls by age; Figure S4: Graphical representation of mean body mass values for boys and girls by age; Figure S5: Graphical representation of mean body fat values for boys and girls by age; Figure S6: Graphical representation of mean fat-free mass values for boys and girls by age; Figure S7: Graphical representation of mean handgrip right hand strength values for boys and girls by age; Figure S8: Graphical representation of mean handgrip left hand strength values for boys and girls by age; Figure S9: Graphical representation of mean 5 m sprint values for boys and girls by age; Figure S10: Graphical representation of mean 10 m sprint values for boys and girls by age; Figure S11: Graphical representation of mean 20 m sprint values for boys and girls by age; Figure S12: Graphical representation of mean 30 m sprint values for boys and girls by age; Figure S13: Graphical representation of mean 40 m sprint values for boys and girls by age; Figure S14: Graphical representation of mean sit-ups values for boys and girls by age; Figure S15: Graphical representation of mean 3 kg seated medicine ball throw values for boys and girls by age; Figure S16: Graphical representation of mean 3 kg seated medicine ball throw right arm values for boys and girls by age; Figure S17: Graphical representation of mean 3 kg seated medicine ball throw left arm values for boys and girls by age; Figure S18: Graphical representation of mean standing long jump values for boys and girls by age; Figure S19: Graphical representation of mean squat jump values for boys and girls by age; Figure S20: Graphical representation of mean countermovement jump values for boys and girls by age; Figure S21: Graphical representation of mean RSI values for boys and girls by age; Figure S22: Graphical representation of mean 1000 m values for boys and girls by age.
