# Supplementary material for: EXcellence and PERformance in Track and Field (EXPERT)—A Mixed-Longitudinal Study on Growth, Biological Maturation, Performance, and Health in Young Athletes: Baseline Results (Part 2)
Source: J Funct Morphol Kinesiol. 2026 Jan 30;11(1):61. doi: 10.3390/jfmk11010061 (PMC12922135; doi:10.3390/jfmk11010061)
Supplement: Supplementary file 1 [file jfmk-11-00061-s001.zip › Supplementary File S1.pdf]

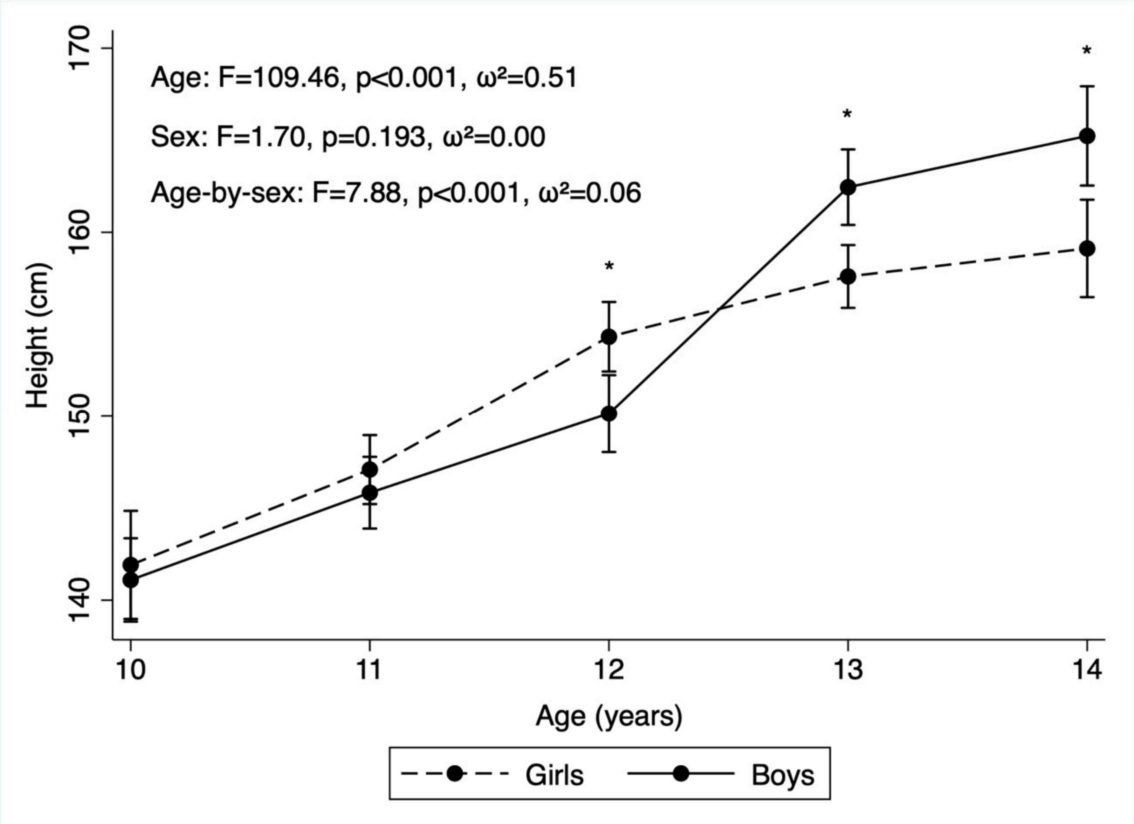

* p≤0.05; • = EM – expected means; bars = SE – standard errors.

Figure S1. Graphical representation of mean height values for boys and girls by age.

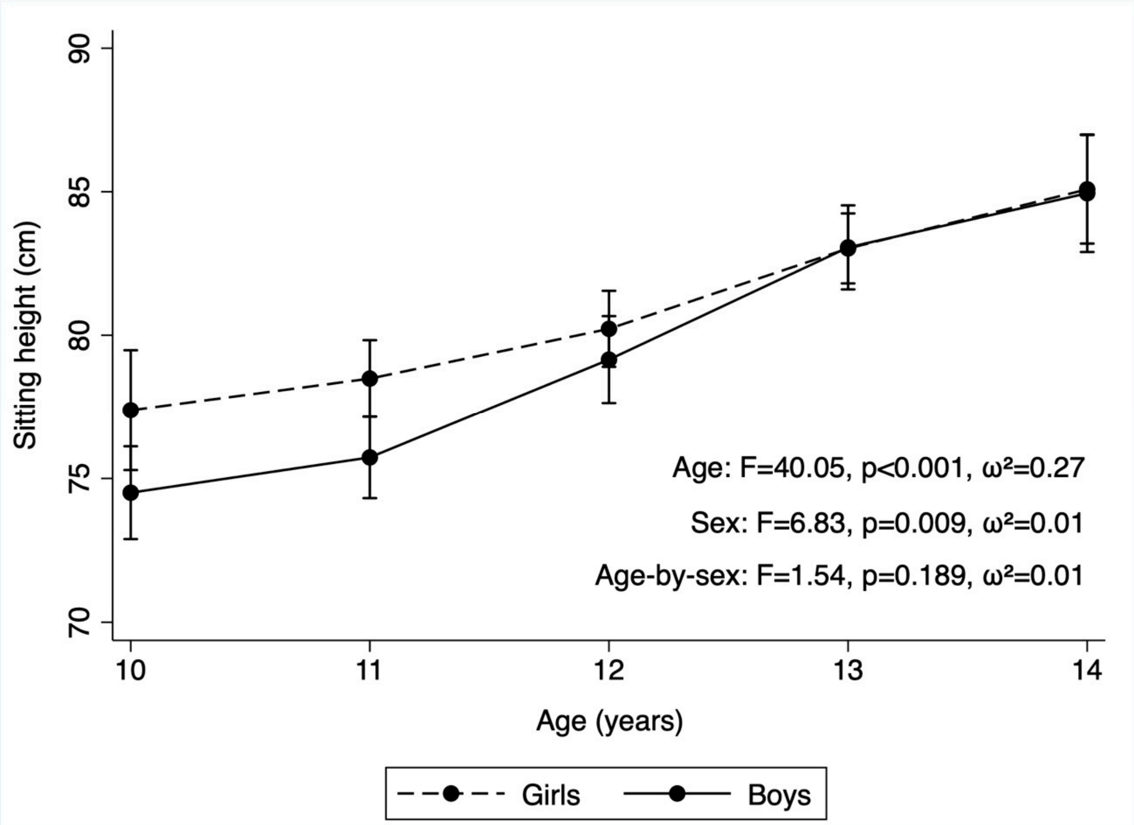

• = EM – expected means; bars = SE – standard errors.

Figure S2. Graphical representation of mean sitting height values for boys and girls by age.

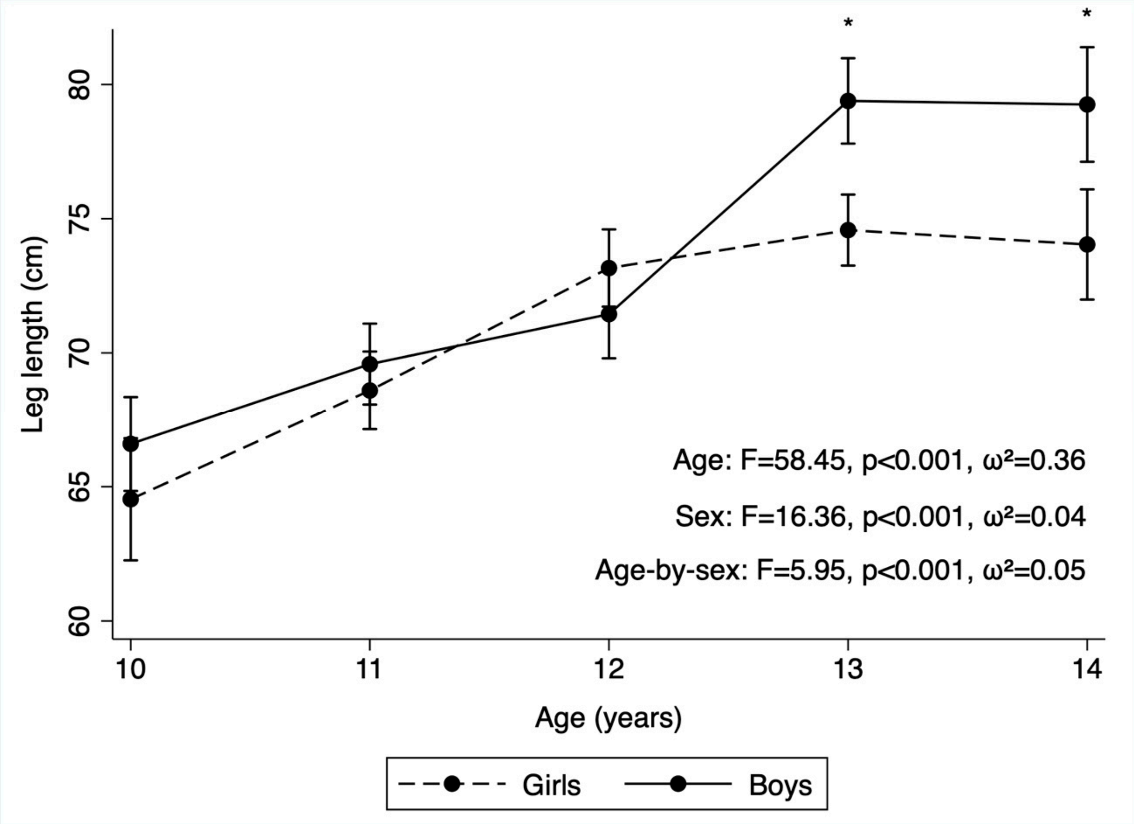

* p≤0.05; • = EM – expected means; bars = SE – standard errors.

Figure S3. Graphical representation of mean leg length values for boys and girls by age.

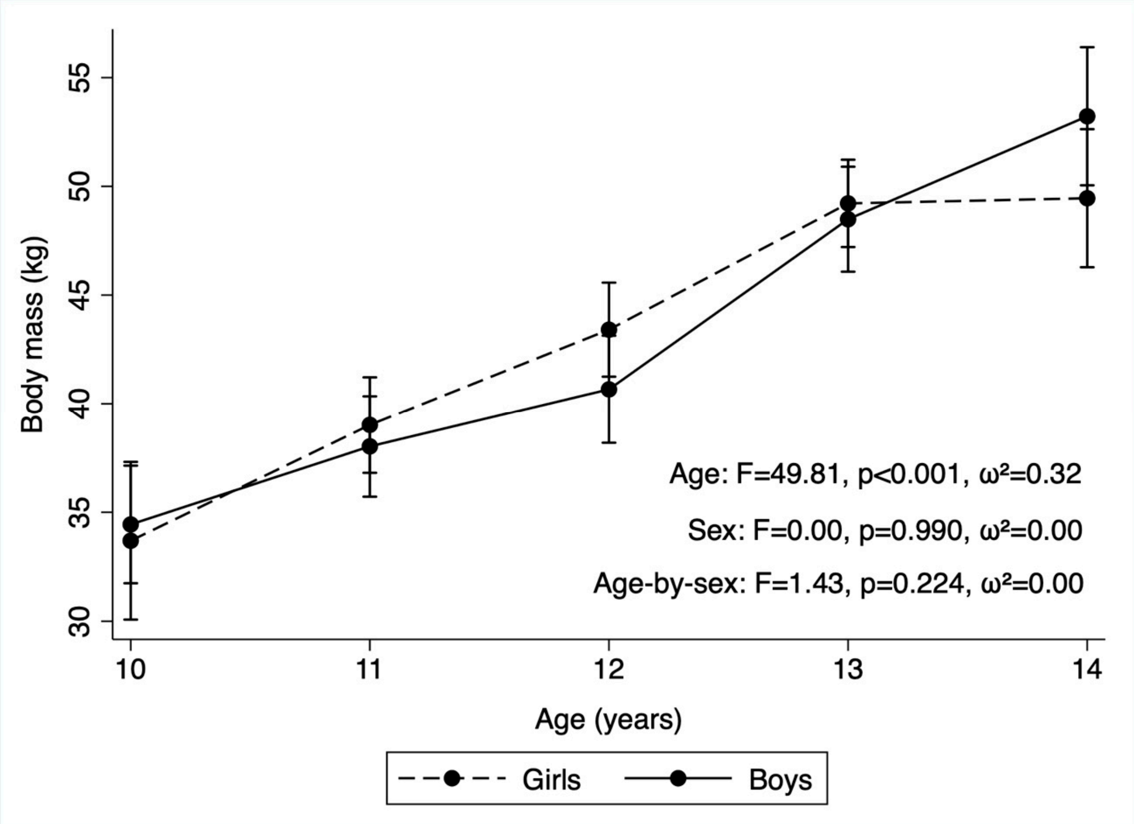

• = EM – expected means; bars = SE – standard errors.

Figure S4. Graphical representation of mean body mass values for boys and girls by age.

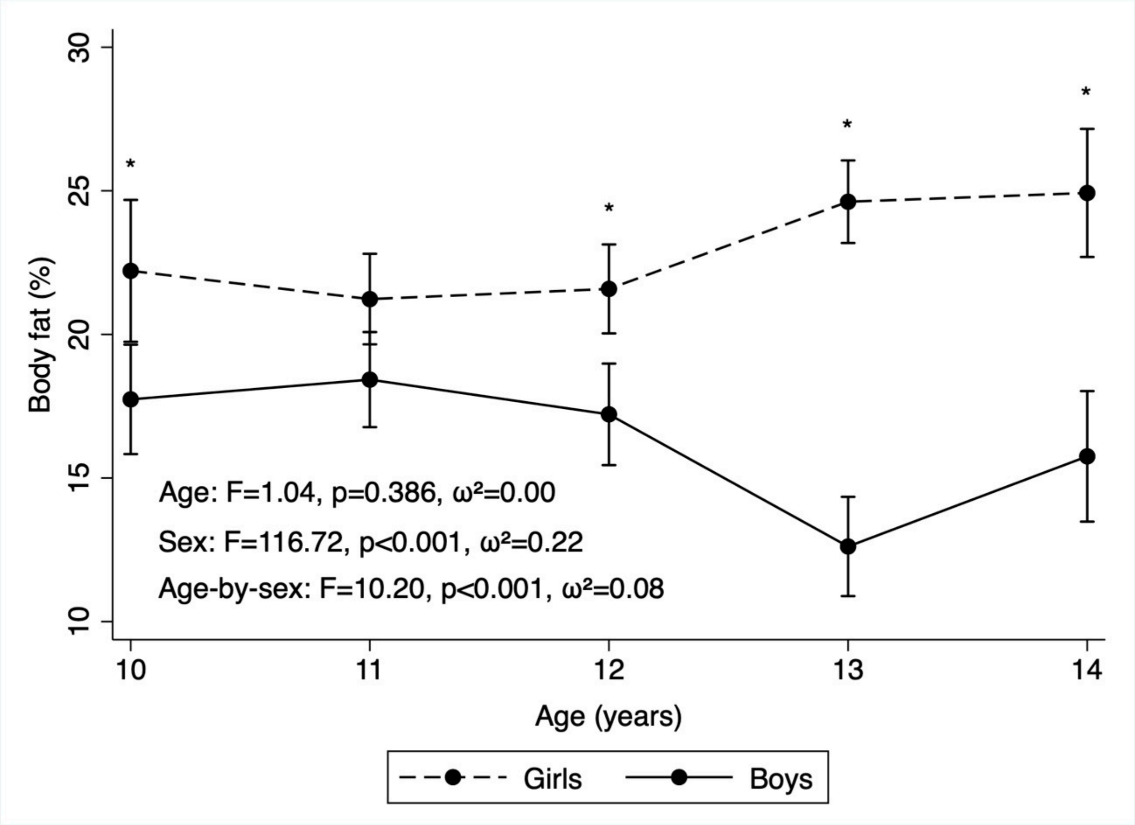

* p≤0.05; • = EM – expected means; bars = SE – standard errors.

Figure S5. Graphical representation of mean body fat values for boys and girls by age.

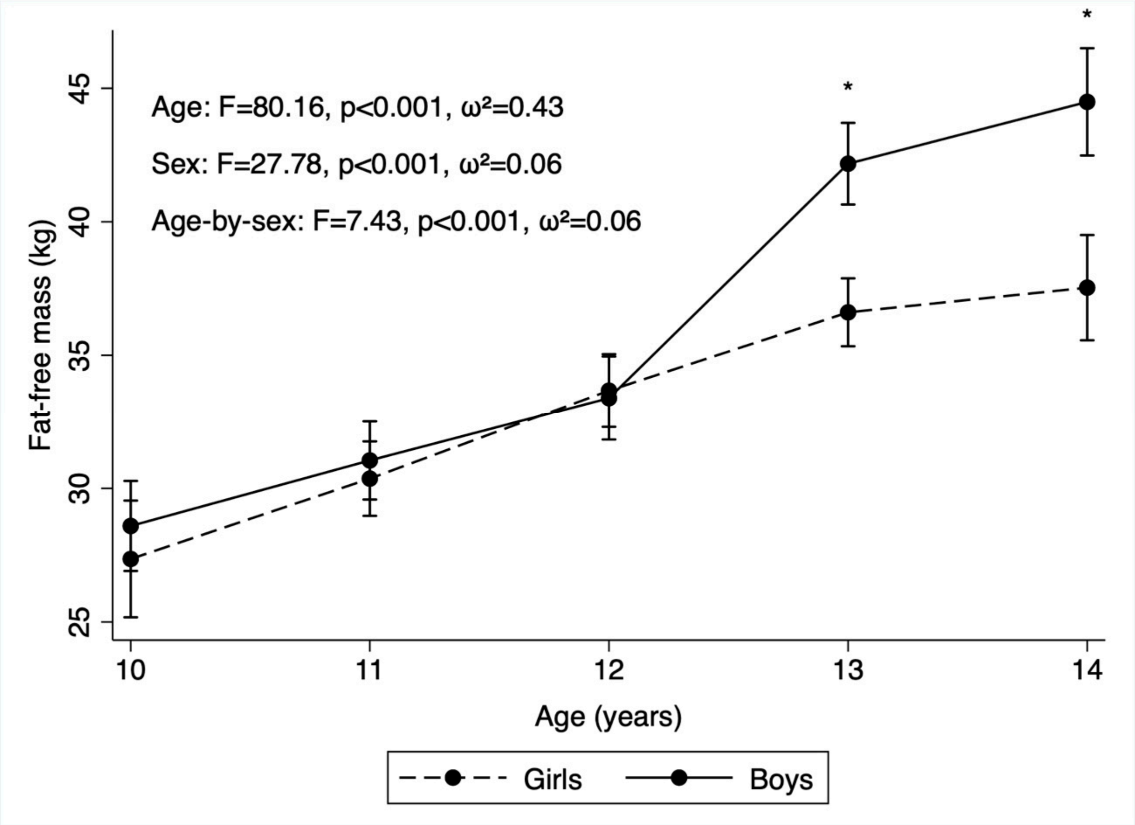

* p≤0.05; • = EM – expected means; bars = SE – standard errors

Figure S6. Graphical representation of mean fat-free mass values for boys and girls by age.
