# Supplementary material for: EXcellence and PERformance in Track and Field (EXPERT)—A Mixed-Longitudinal Study on Growth, Biological Maturation, Performance, and Health in Young Athletes: Baseline Results (Part 2)
Source: J Funct Morphol Kinesiol. 2026 Jan 30;11(1):61. doi: 10.3390/jfmk11010061 (PMC12922135; doi:10.3390/jfmk11010061)
Supplement: Supplementary file 1 [file jfmk-11-00061-s001.zip › Supplementary File S2.pdf]

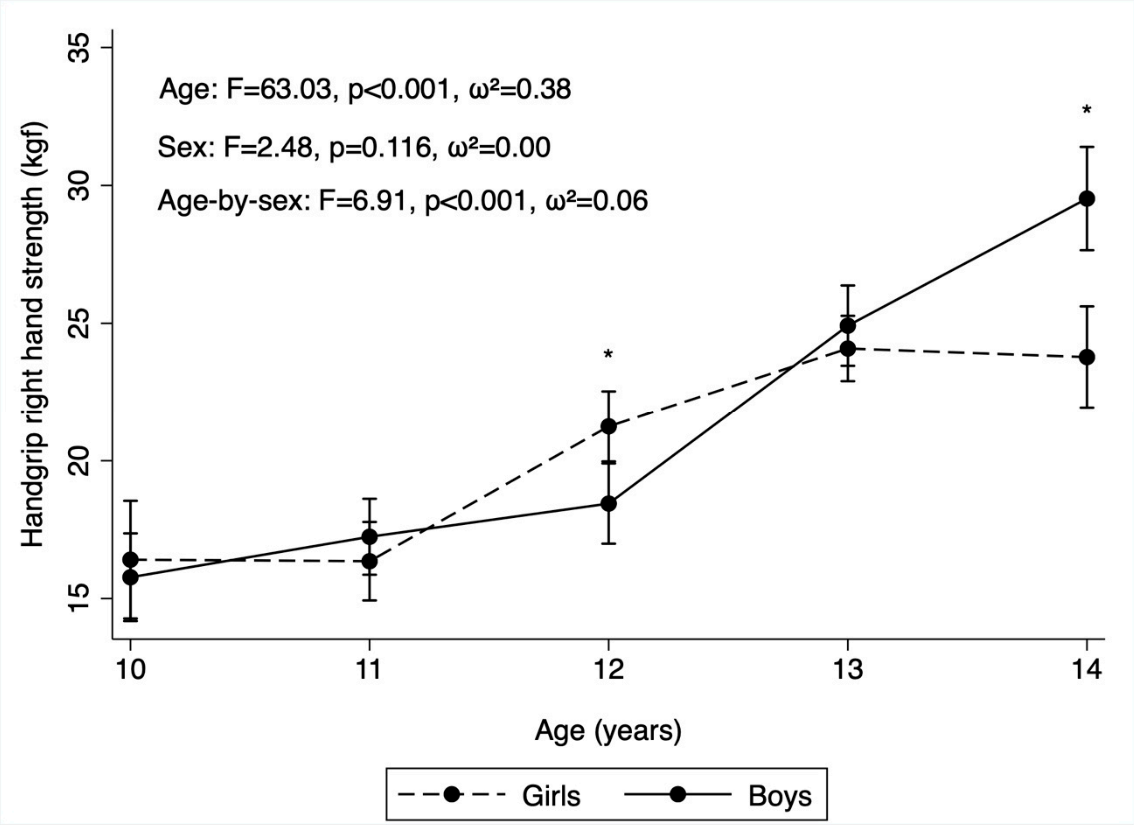


* p≤0.05; • = EM – expected means; bars = SE – standard errors.

**Figure S7.** Graphical representation of mean handgrip right hand strength values for boys and girls by age.

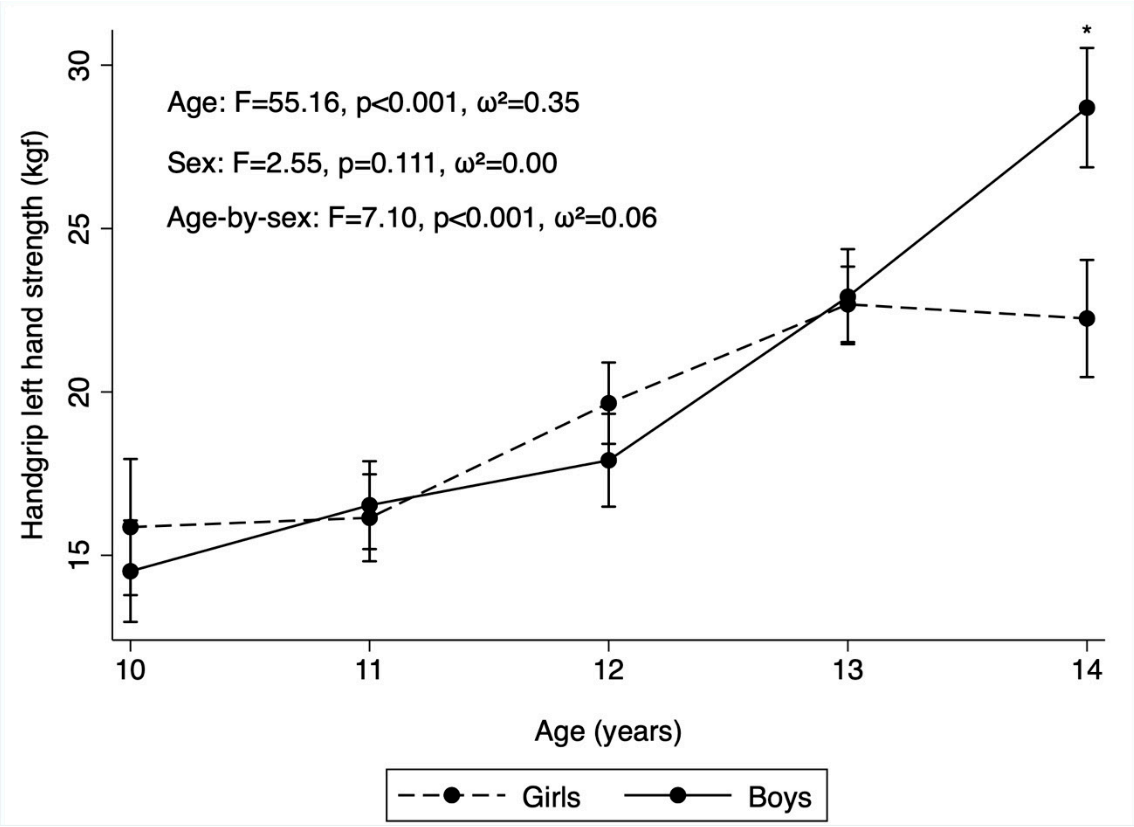


* p≤0.05; • = EM – expected means; bars = SE – standard errors.

**Figure S8.** Graphical representation of mean handgrip left hand strength values for boys and girls by age.

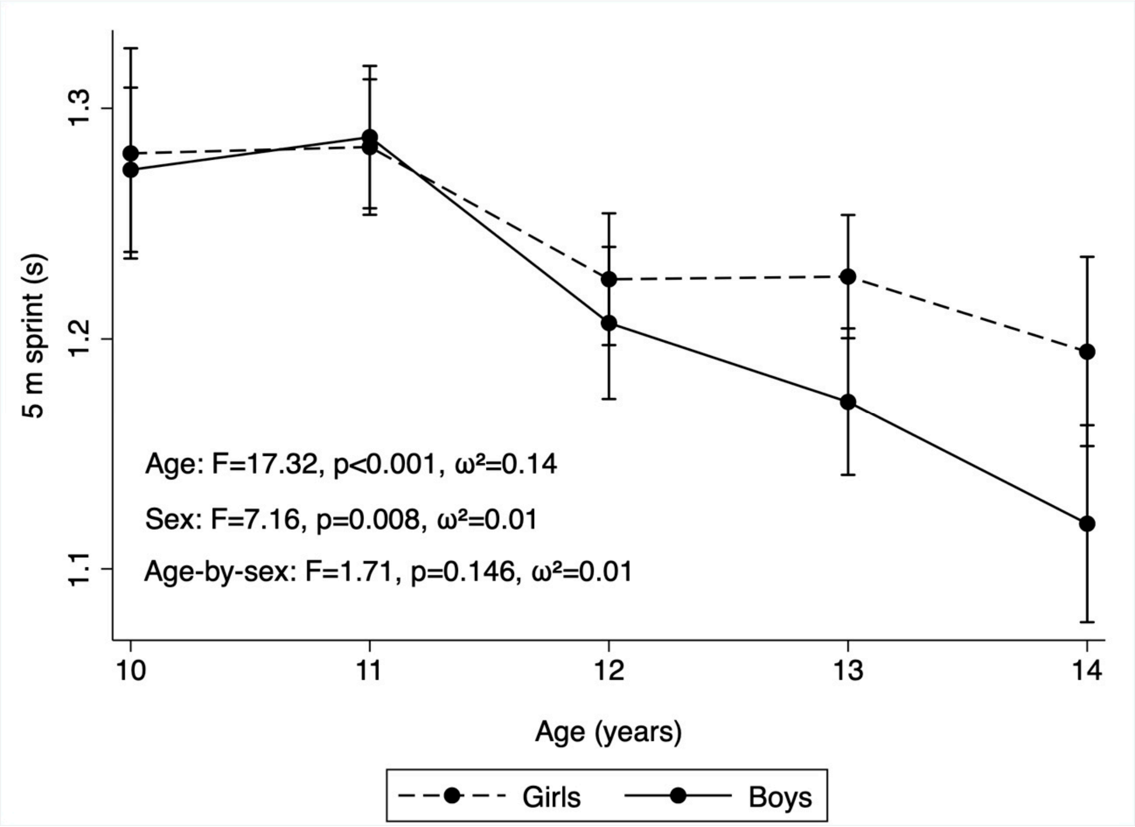


• = EM – expected means; bars = SE – standard errors.

**Figure S9.** Graphical representation of mean 5 m sprint values for boys and girls by age.

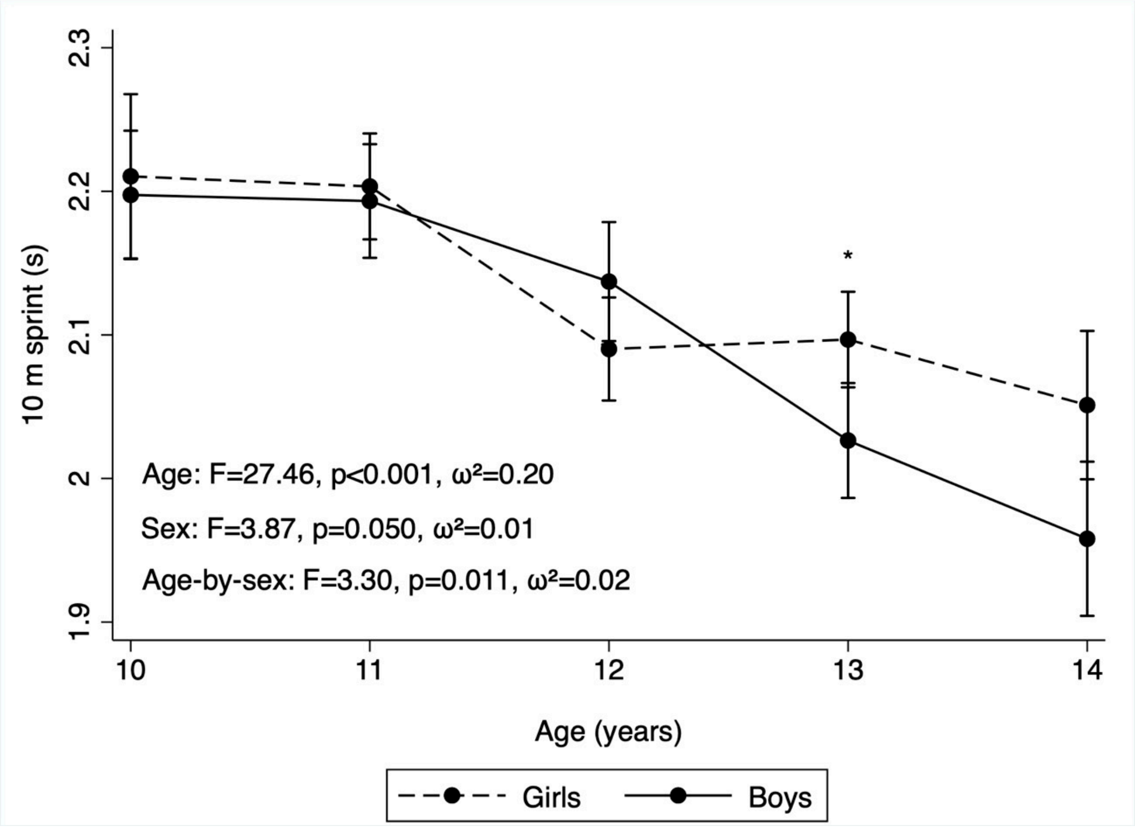


* p≤0.05; • = EM – expected means; bars = SE – standard errors.

**Figure S10.** Graphical representation of mean 10 m sprint values for boys and girls by age.

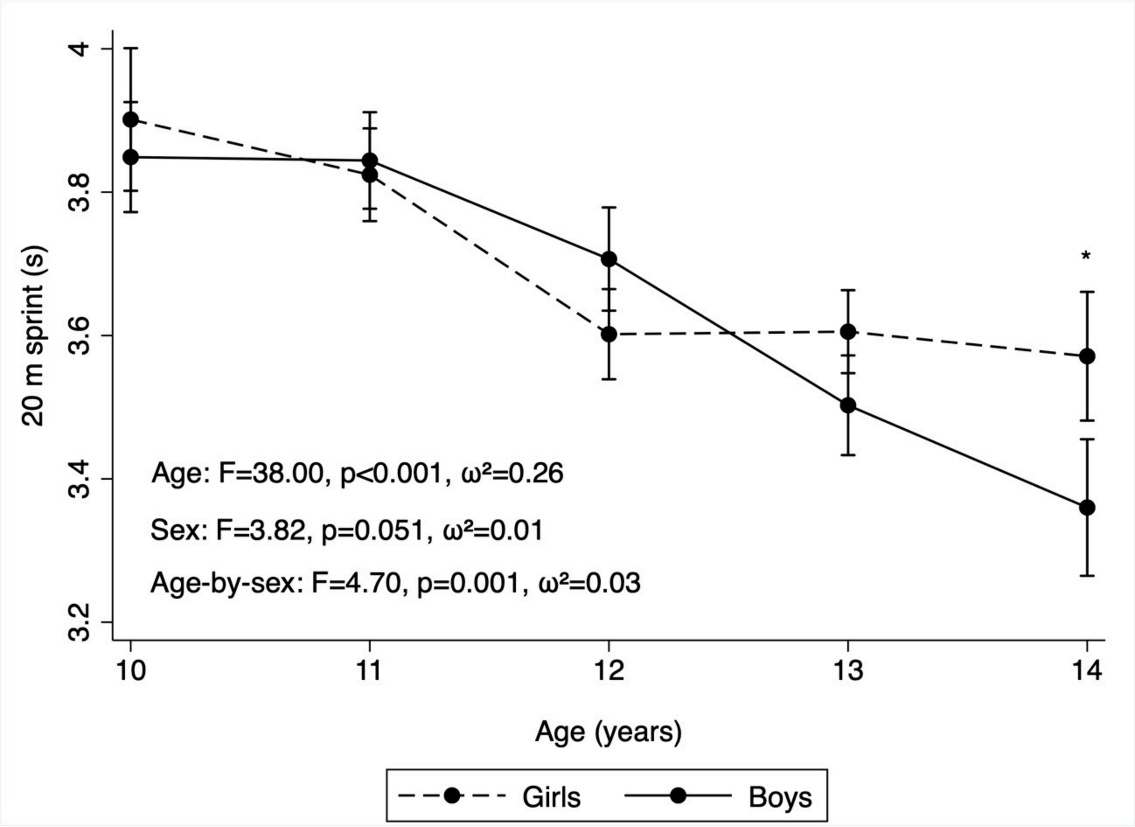


* p≤0.05; • = EM – expected means; bars = SE – standard errors.

**Figure S11.** Graphical representation of mean 20 m sprint values for boys and girls by age.

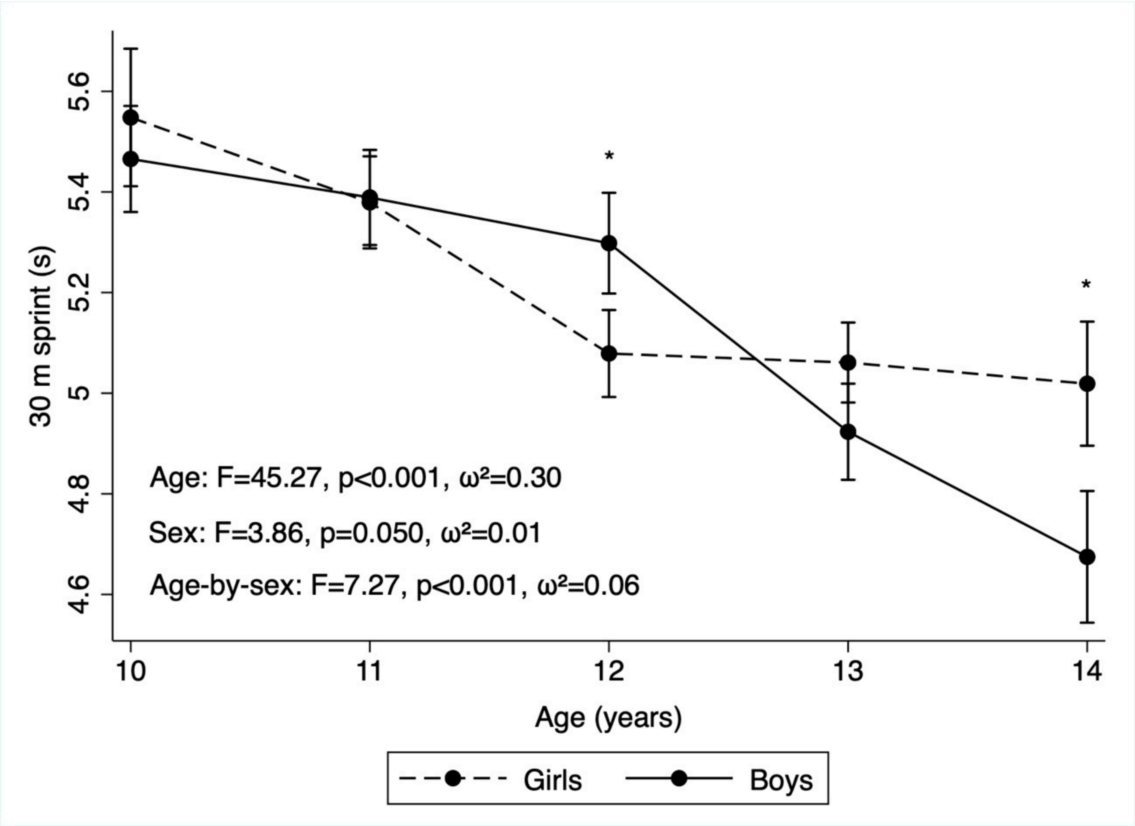


* p≤0.05; • = EM – expected means; bars = SE – standard errors.

**Figure S12.** Graphical representation of mean 30 m sprint values for boys and girls by age.

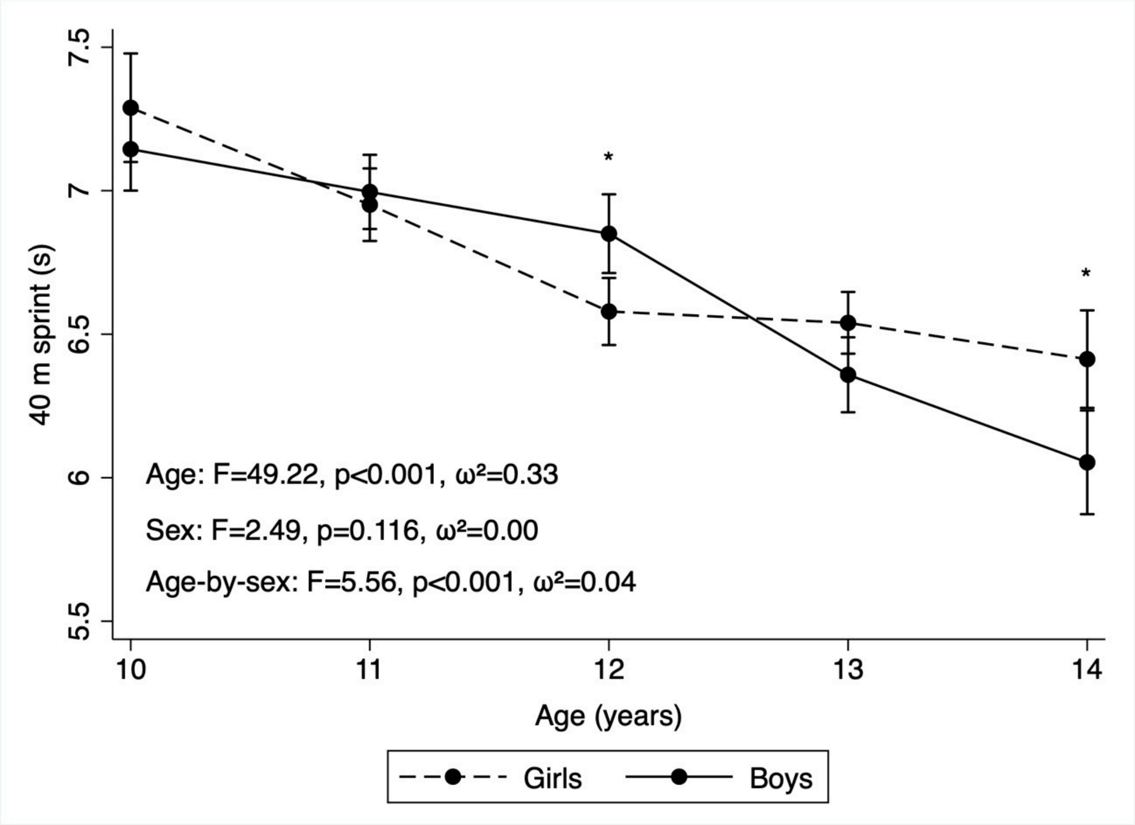


* p≤0.05; • = EM – expected means; bars = SE – standard errors.

**Figure S13.** Graphical representation of mean 40 m sprint values for boys and girls by age.

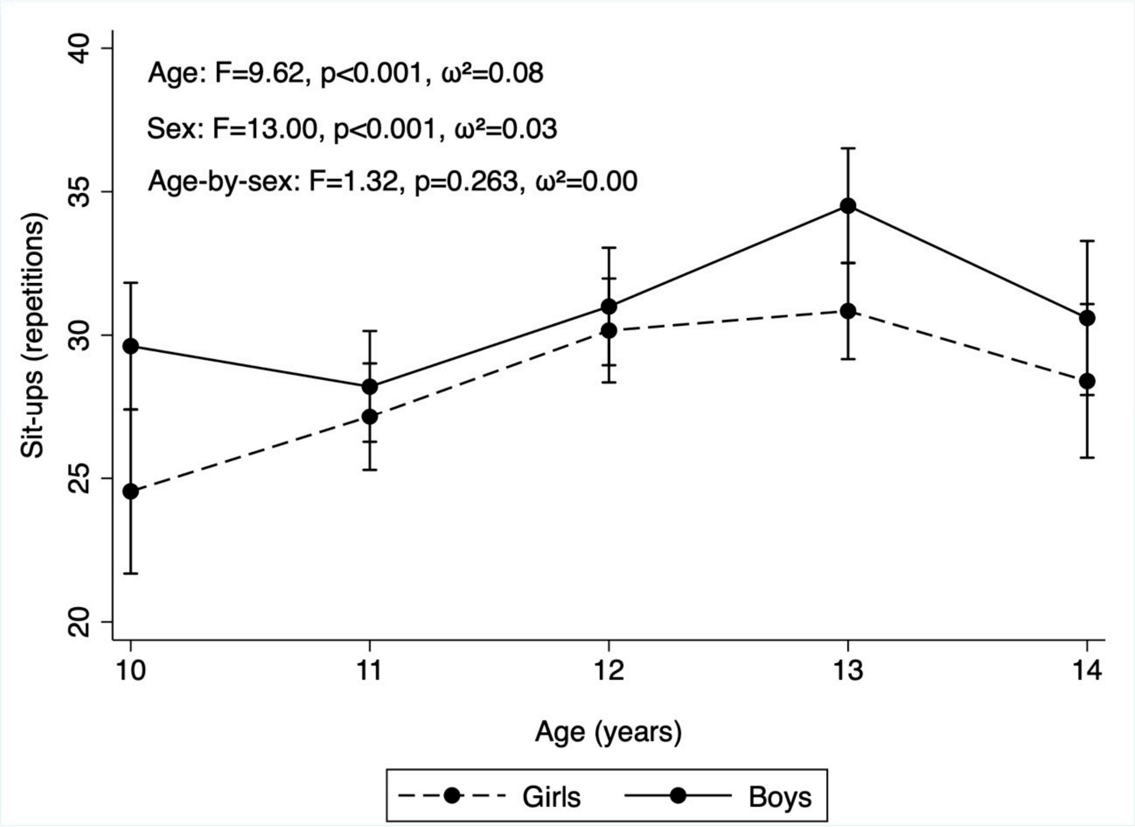


• = EM – expected means; bars = SE – standard errors.

**Figure S14.** Graphical representation of mean sit-ups values for boys and girls by age.

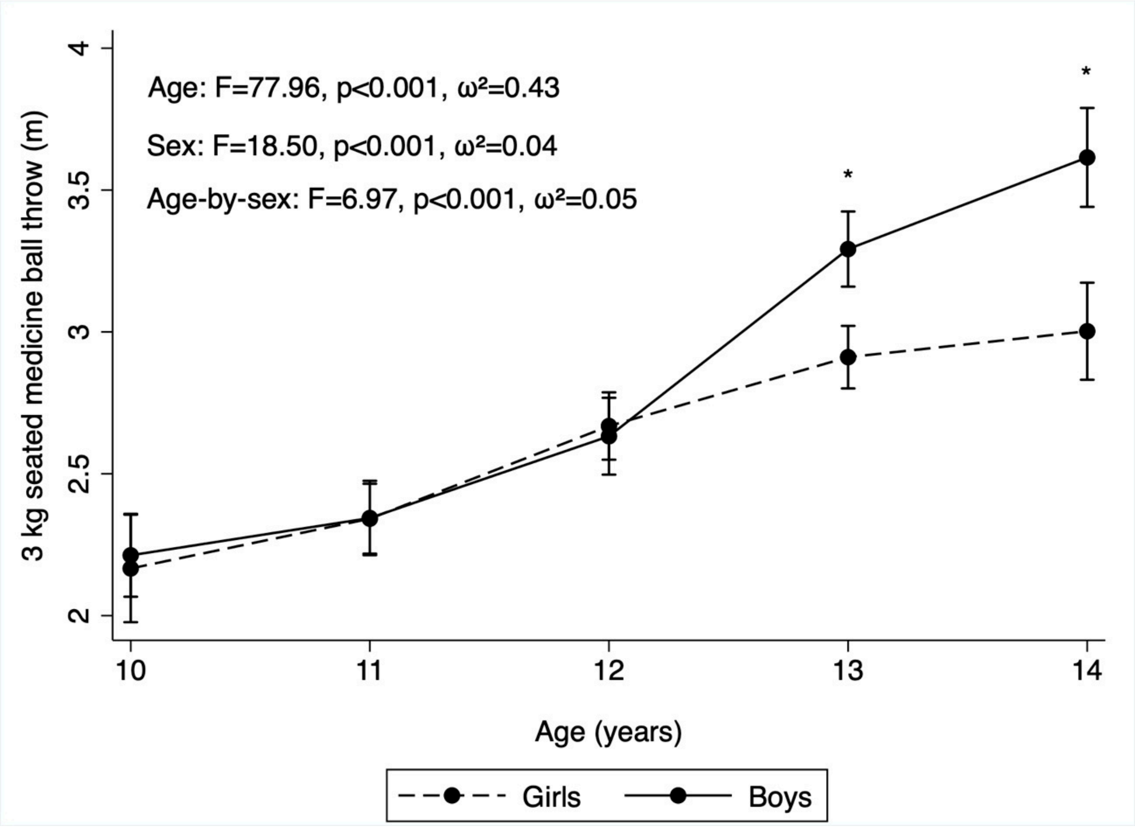


* p≤0.05; • = EM – expected means; bars = SE – standard errors.

**Figure S15.** Graphical representation of mean 3 kg seated medicine ball throw values for boys and girls by age.

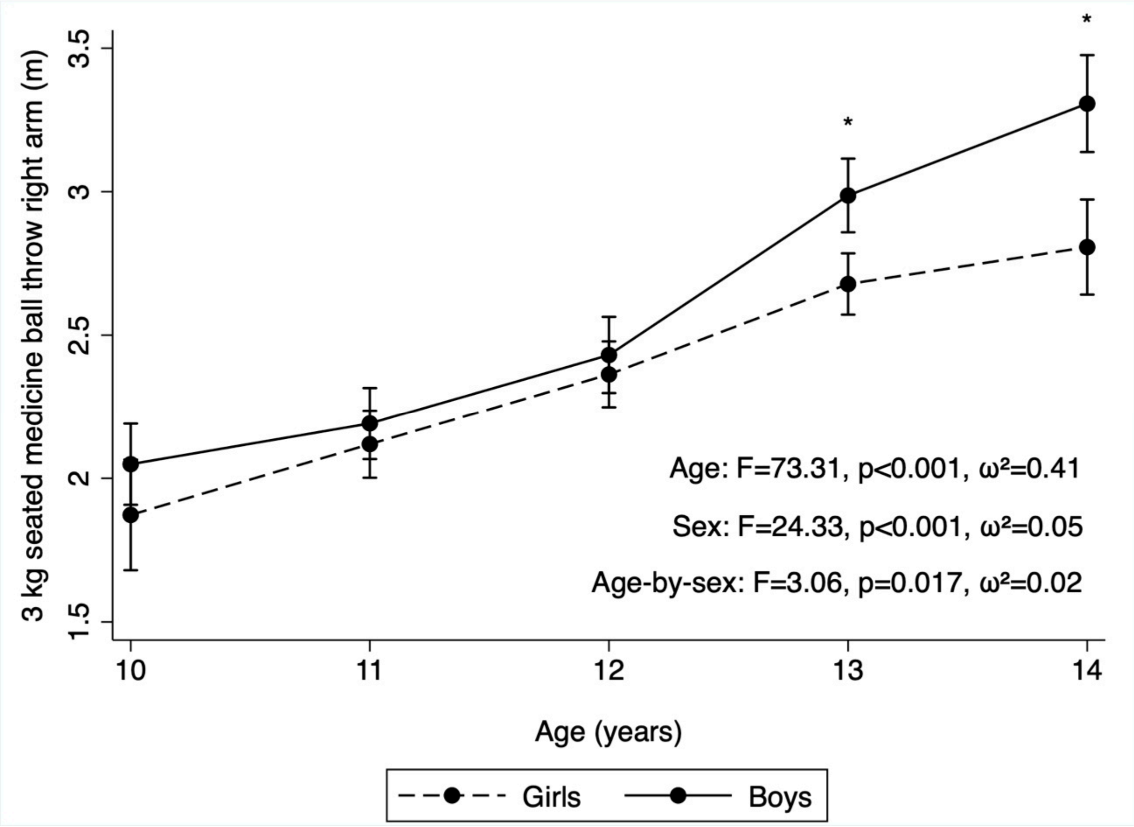


* p≤0.05; • = EM – expected means; bars = SE – standard errors.

**Figure S16.** Graphical representation of mean 3 kg seated medicine ball throw right arm values for boys and girls by age.

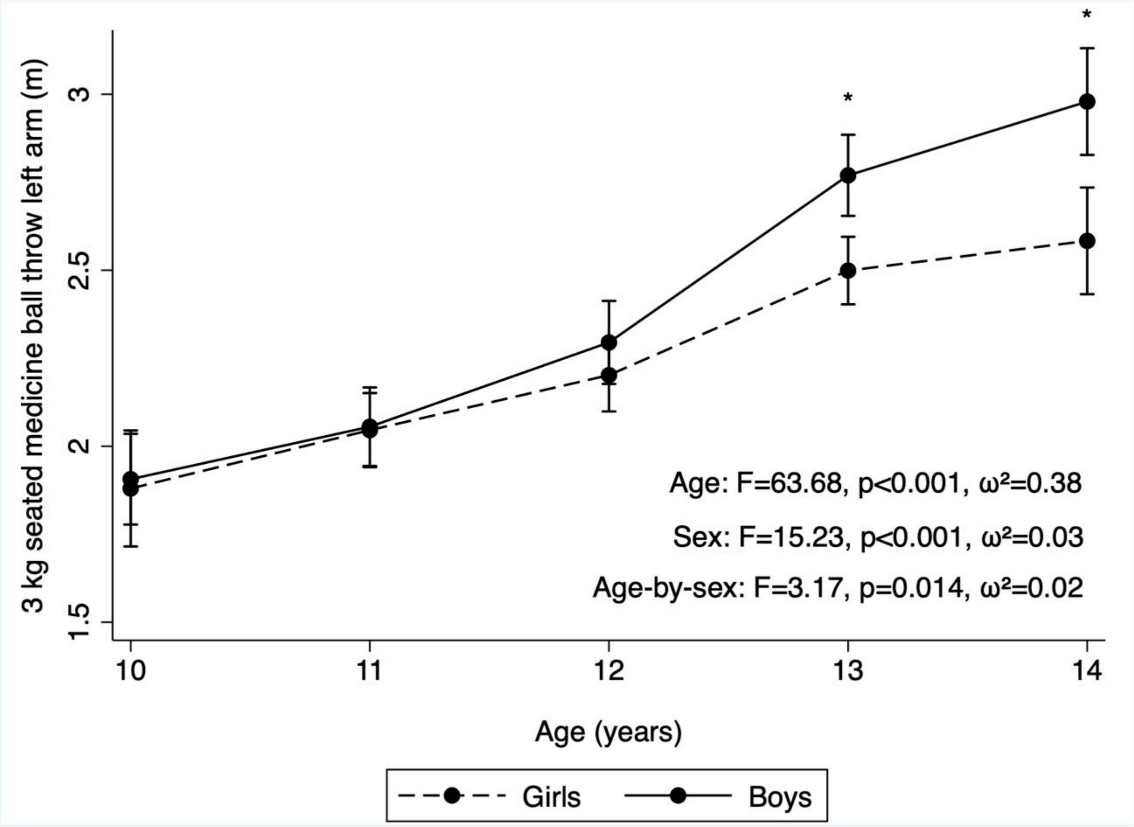


* p≤0.05; • = EM – expected means; bars = SE – standard errors.

**Figure S17.** Graphical representation of mean 3 kg seated medicine ball throw left arm values for boys and girls by age.

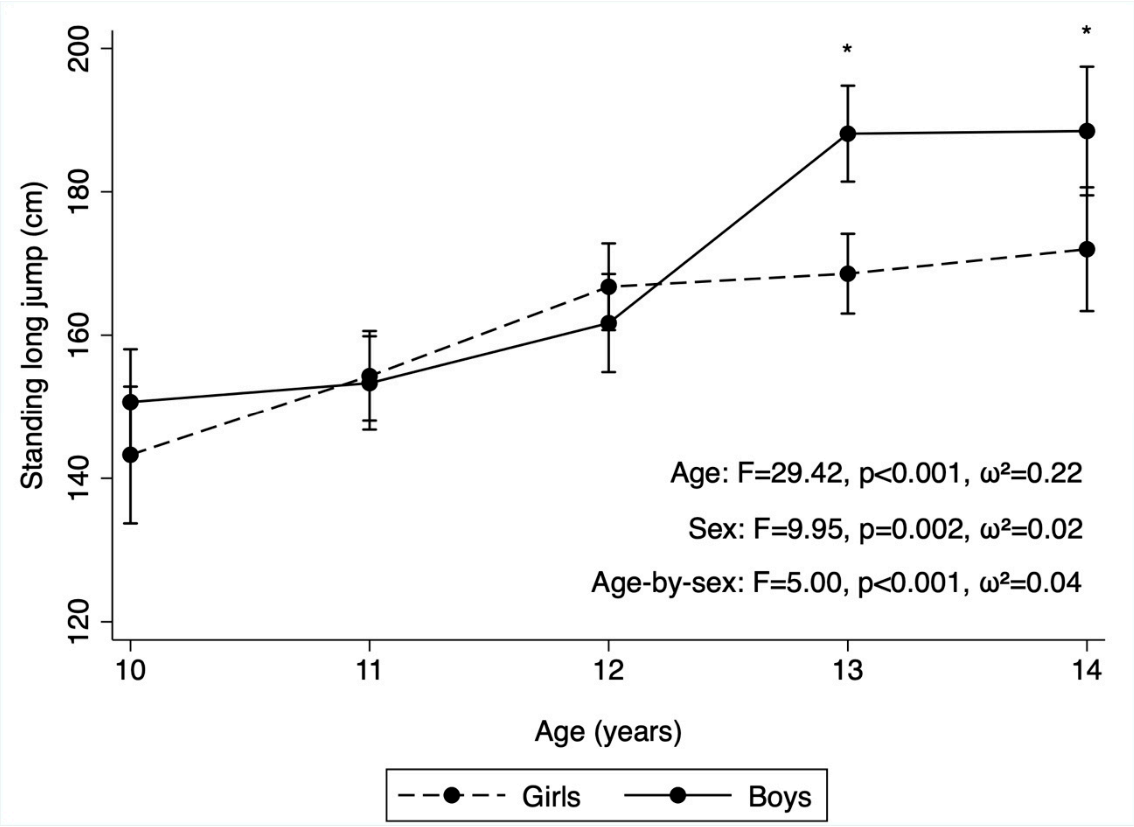


* p≤0.05; • = EM – expected means; bars = SE – standard errors.

**Figure S18.** Graphical representation of mean standing long jump values for boys and girls by age.

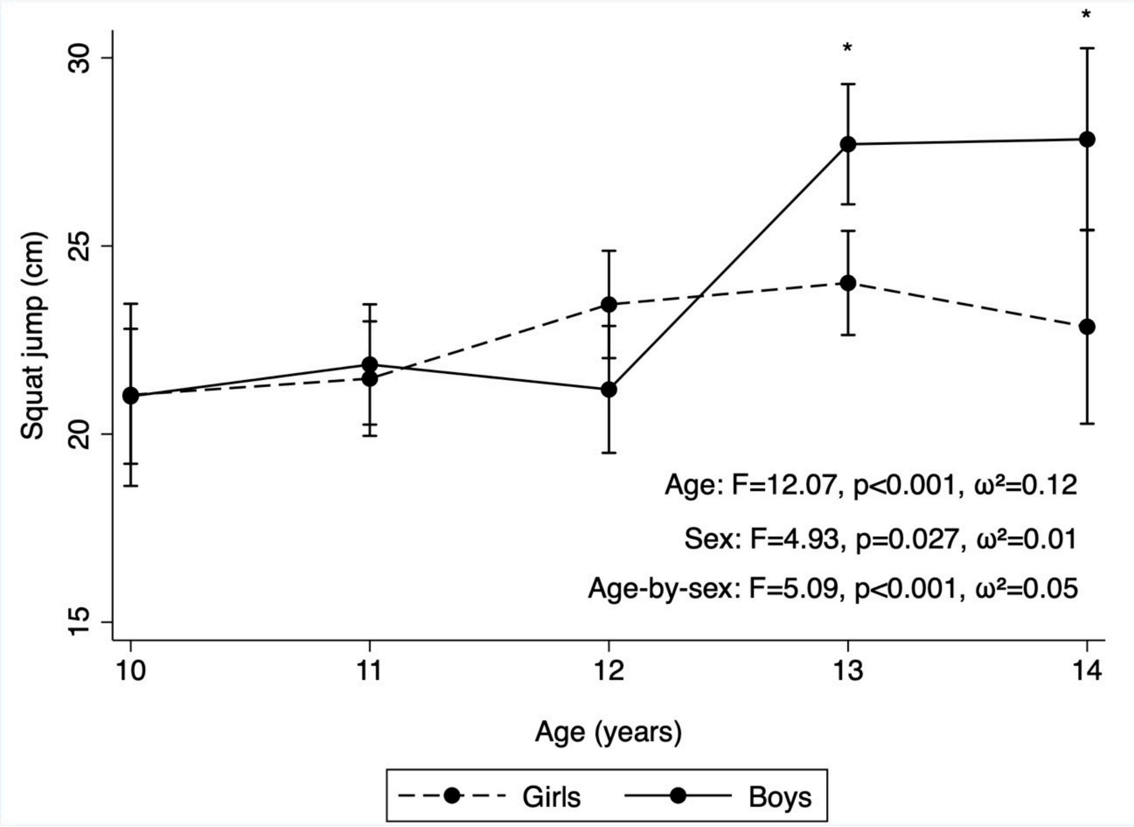


* p≤0.05; • = EM – expected means; bars = SE – standard errors.

**Figure S19.** Graphical representation of mean squat jump values for boys and girls by age.

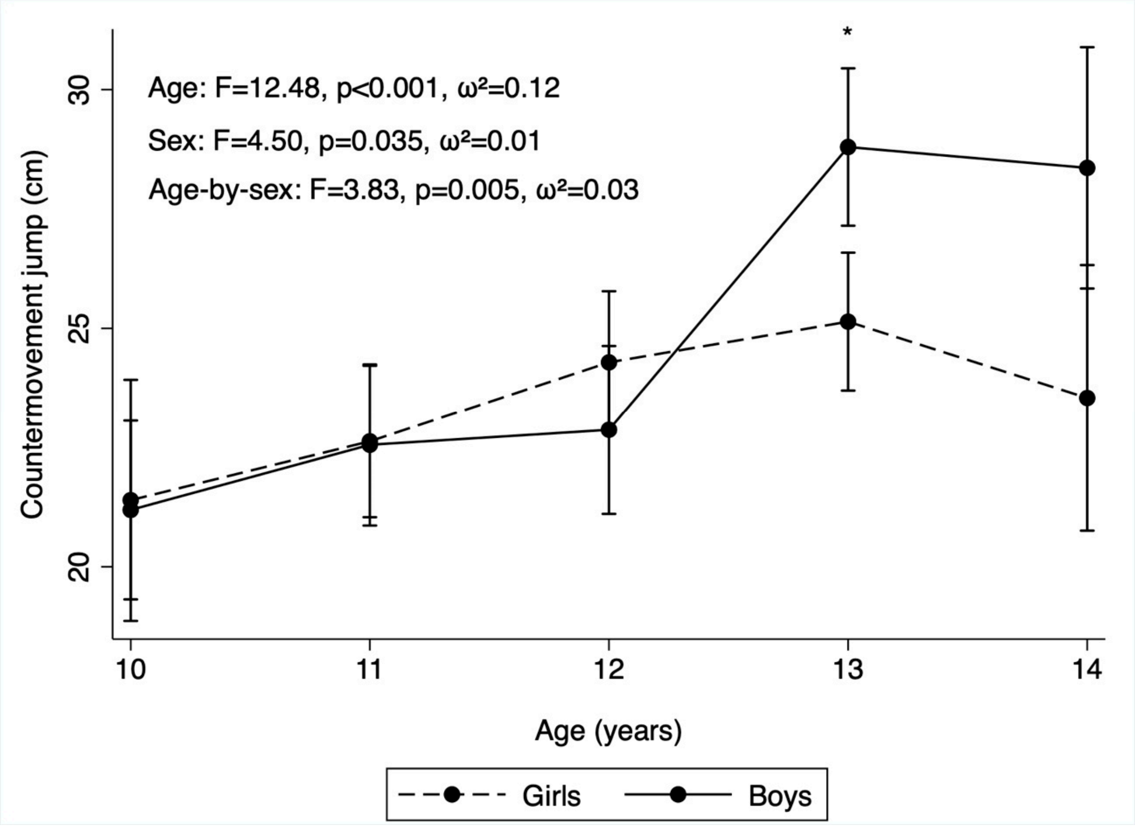


* p≤0.05; • = EM – expected means; bars = SE – standard errors.

**Figure S20.** Graphical representation of mean countermovement jump values for boys and girls by age.

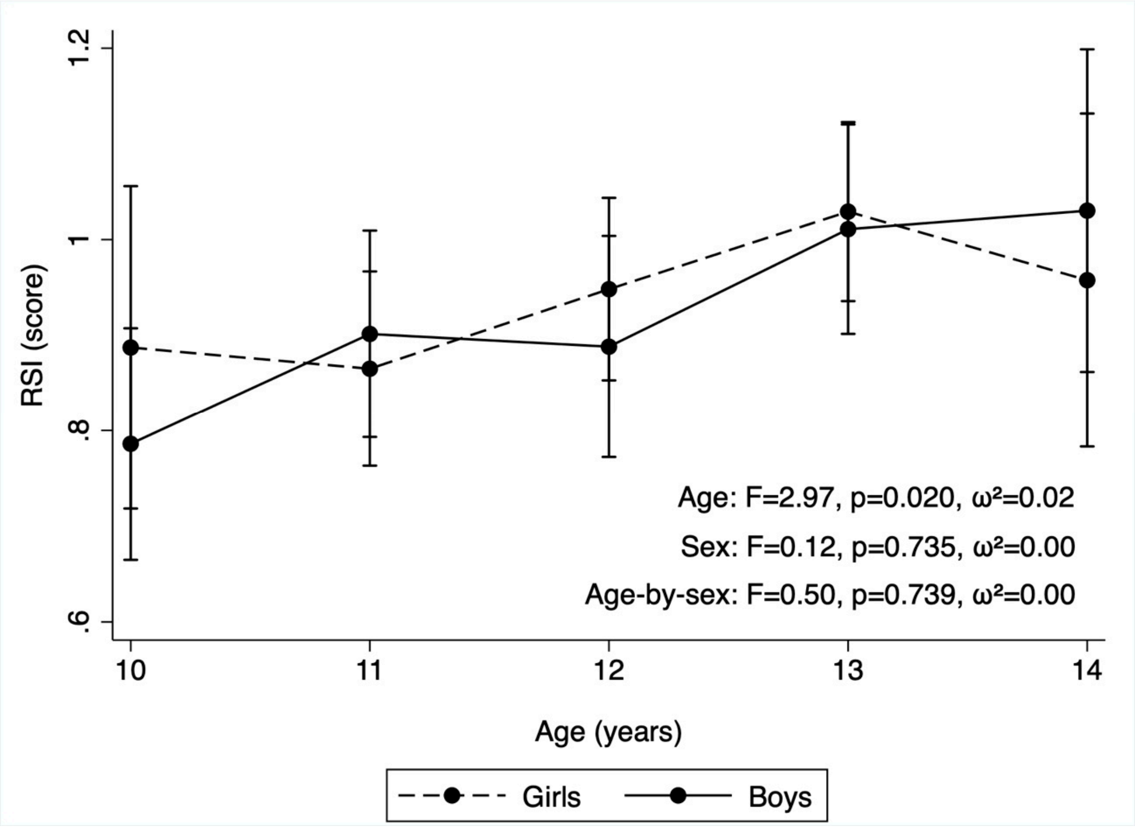


• = EM – expected means; bars = SE – standard errors.

**Figure S21.** Graphical representation of mean RSI values for boys and girls by age.

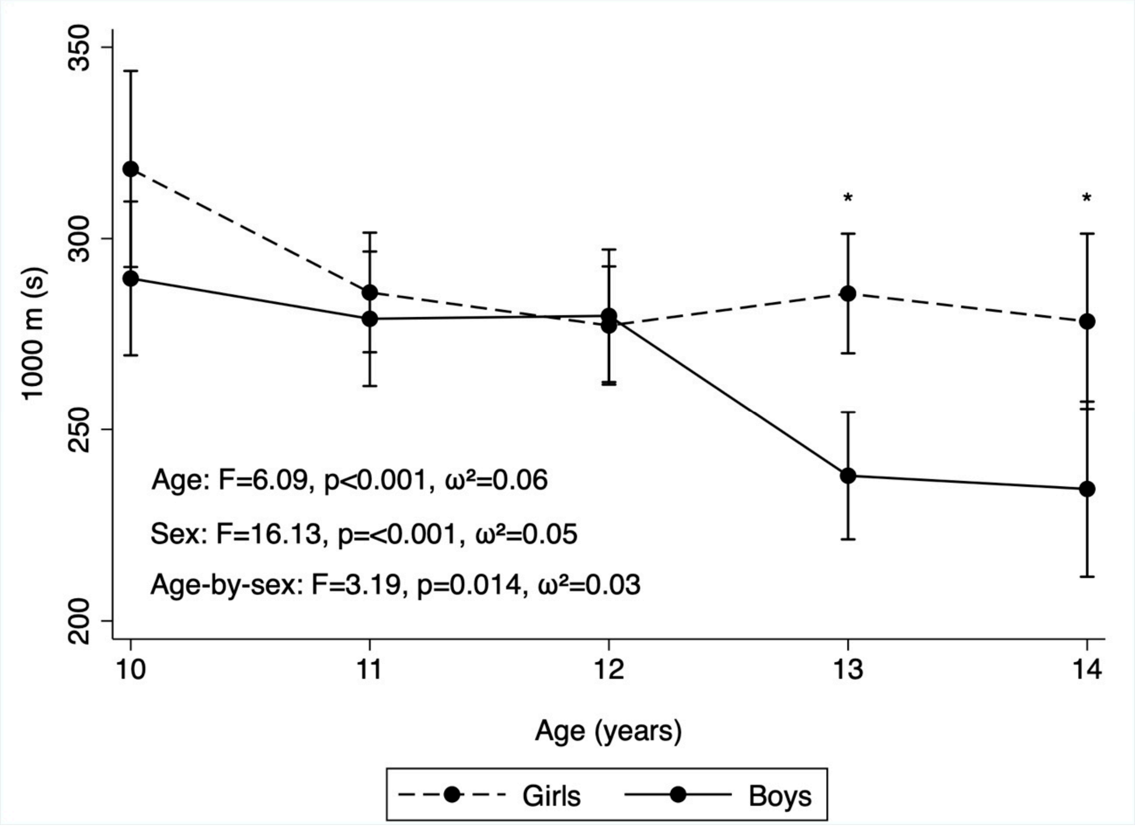


* p≤0.05; • = EM – expected means; bars = SE – standard errors.

**Figure S22.** Graphical representation of mean 1000 m values for boys and girls by age.
